# Designed coiled-coil peptide nanoparticles with tunable self-assembly: distinct ordered nanostructures *via* nonnatural side chain modification and electrostatic screening†

**DOI:** 10.1039/d5sm00972c

**Published:** 2025-12-01

**Authors:** Amanda L. McCahill, Tianren Zhang, Jacob Schwartz, Yi Shi, Christopher J. Kloxin, Jeffery G. Saven, Darrin J. Pochan

**Affiliations:** a Department of Materials Science and Engineering, University of Delaware Newark DE 19716 USA pochan@udel.edu; b Department of Chemical and Biomolecular Engineering, University of Delaware Newark DE 19716 USA; c Department of Chemistry, University of Pennsylvania Philadelphia PA 19104 USA

## Abstract

Peptide ‘bundlemers’ are homotetrameric coiled-coils that form discrete cylindrical nanoparticles in aqueous solution. The stability of the coiled-coil design enables selective modification of exterior residues to program interparticle interactions. We designed a single charge-type (SC) bundlemer, containing only positive ionizable groups on its exterior, with allyloxycarbonyl (alloc)-protected lysine positioned to direct hydrophobic interparticle assembly. SC bundlemers with no alloc functionalization form liquid crystalline phases through end-to-end stacking of nanoparticles into chains that are laterally repulsive due to electrostatic interactions. Incorporation of alloc groups into the SC design yielded different, ordered nanostructures depending on solution conditions. Nematic and hexagonal columnar liquid crystals were formed in pure water, where end-to-end particle stacking and lateral repulsion dominated. In contrast, ordered nanoporous lattices were formed in the presence of salt, where electrostatic screening revealed alloc-driven, lateral hydrophobic interactions coupled with particle end-to-end stacking. Uniquely, the lattices exhibited structure factor signatures identical to those of the previously observed in alloc-modified mixed charge bundlemers, highlighting how precise, protein-like spatial display of desired chemical functionality enables targeted interparticle interactions and tunable nanostructure formation.

## Abstract 2

Peptide ‘bundlemers’ are homotetrameric coiled-coils that form discrete cylindrical particles in aqueous solution. Depending on solution conditions, bundlemers can form different ordered nanostructures from liquid crystals to nanoporous lattices.

## Graphical abstract

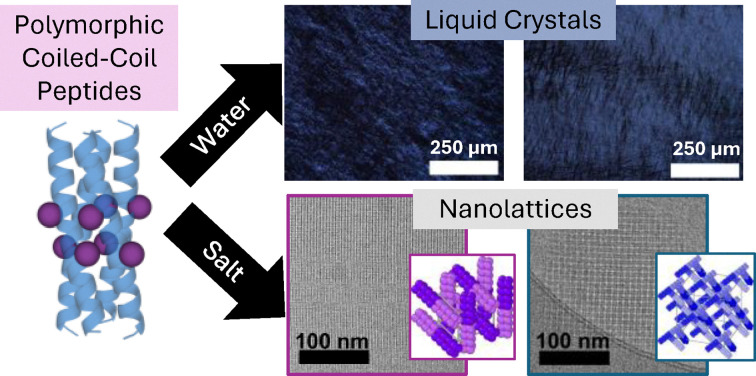

## Introduction

The creation of materials with targeted nanostructure through programmable self-assembly is accomplished *via* the design of molecules with tunable interactions, enabling the bottom-up construction of complex material systems. Self-assembled materials have been engineered to be stimuli-responsive using various molecular building blocks, including colloids,^1,2^ block copolymers,^3–5^ amphiphiles,^6,7,13^ and proteins.^8–12^ Modes of stimuli for responsive behavior include thermal, light, pH, electrical, physical, mechanical, and chemical triggers.^8–10,13,14^ Protein and peptide self-assembled materials in particular have been used in stimuli responsive applications, including drug delivery,^8–10,12,15^ tissue engineering,^8,9,12^ biocatalysis,^8^ sensing,^8,10,12^ and coatings.^8^ More sophisticated systems integrate dual- and multi-stimuli responsiveness, offering temporal control over nanostructure and enabling specific applications in targeted drug delivery and release.^4,16–18^ The ability to design new molecules and self-assembly mechanisms with responsive behavior is critical for the development of the next generation of smart materials.

A common design strategy for stimuli-responsive materials is the order-to-disorder transition, where external stimuli destabilize supramolecular structures.^9^ The transition is exemplified by proteins that unfold from ordered, functional globular states into disordered, nonfunctional chains upon exposure to changes in temperature, pH, or salt concentration.^8^ Supramolecular protein assemblies can also undergo order-to-disorder transitions. For example, proteins with a lower critical solution temperature (LCST) self-assembly above their LCST but are dispersed and soluble below it,^8,10^ while those with an upper critical solution temperature (UCST) behave oppositely with respect to temperature.^19,20^ Control over the critical solution temperature can be achieved *via* protein design, varying the size and structure of hydrophobic and hydrophilic regions.^9^ Similar temperature-dependent order-to-disorder transitions occur in block copolymers^5^ that lose bulk nanostructure upon heating and in thermotropic liquid crystals that transition from ordered liquid crystalline phases to isotropic liquids.^21,22^ The ability to destabilize supramolecular structures using external stimuli enables these order-to-disorder transitions, providing a pathway for controllable structure formation or denaturation as well as reversible phase transitions.

In contrast, single molecules or nanoparticles designed to allow controlled assembly into two or more distinct ordered phases are relatively uncommon. Block copolymers are the most prominent example, where tuning block composition and relative block length can direct specific nanostructure formation. However, by changing solution conditions; such as concentration, temperature, or pH; order-to-order transitions can be induced between solution micellar, membranous, or bulk phases.^5,23–26^ Another example is with synthetic colloidal crystals that can be designed to transition between different ordered crystalline phases with tunable interparticle interactions, dependent on temperature and volume fraction.^2^ Some proteins, such as poly-l-lysine, undergo a secondary structure transition between an α-helix or β-sheet under specific pH and thermal conditions,^27,28^ a process relevant to fibrillization and neurodegenerative diseases.^8^ Liquid crystals also undergo order-to-order phase transitions between distinct mesophases, such as smectic and nematic, upon changes in concentration and temperature.^22,29^ Inspired by the different, multiple ordered phases possible in the examples above, we sought to design peptides capable of hierarchical assembly into coiled-coil nanoparticles that, in turn, form two different, ordered structures depending on solution conditions. Accordingly, it is essential to understand the peptide sequence-hierarchical structure relationship for successful formation of multiple, targeted, ordered structures.

Bundlemers are computationally designed, homotetrameric coiled-coils made from 29 amino acid peptides that assemble into cylindrical nanoparticles approximately 2 nm in diameter and 4 nm in length.^30,31^ Variants have been computationally designed to assemble with either parallel and antiparallel peptide orientation,^32,33^ different peptide lengths (*e.g.*, 15, 22, 29 residues),^34^ mixed-charge sequences (*i.e.*, peptides that contain both basic and acidic residues),^32^ or single-charge sequences (*i.e.*, peptides that contain exclusively acidic or basic residues).^35^ Bundlemers have been modified with peripheral click chemistry functional handles for covalent conjugation into highly rigid nanorods,^36–39^ crosslinked networks,^40^ and temperature-responsive hydrogels.^34^ Through non-covalent interactions, they have also been assembled into electrostatically stabilized porous lattices with specific symmetry,^31^ pH-responsive nanostructures (nanotubes, nanosheets, and needles),^41^ hydrophobic-driven nanoporous lattices,^40^ and end-to-end stacked liquid crystalline phases.^33,35^ Collectively, this toolbox of covalent and physical pathways provides a framework for understanding sequence–structure relationships and highlights how site-specific modification on a bundlemer surface can be exploited to target specific nanostructures.

Previous work showed that hydrophobic allyloxycarbonyl (alloc) modification at specific surface sites in mixed-charge (MC) bundlemers induced assembly into highly ordered, nanoporous lattices.^40^ Separately, single-charge (SC) bundlemers were found to assemble into liquid crystalline phases through end-to-end interparticle stacking with lateral electrostatic repulsion between stacked chains stabilizing nematic liquid crystals and a more highly ordered hexagonal columnar phase at higher concentrations.^35^ Herein, we hypothesized that incorporation of alloc modification into an SC sequence would enable control over two distinct ordered nanostructures, liquid crystalline phases or nanoporous lattices, depending on solution conditions. Specifically, electrostatic screening by salt should suppress lateral repulsion, unmasking alloc-driven hydrophobic interactions and driving lattice formation, while in the absence of salt, electrostatic repulsion should preserve liquid crystalline assembly. By leveraging the stability and chemical addressability of bundlemers, we aim to demonstrate that a single bundlemer design is capable of forming two distinct, ordered nanostructures in response to simple environmental stimuli.

## Results and discussion

Two single-charge (SC) liquid crystal-forming bundlemer sequences, SC+6 and SC+8,^35^ were chosen as parent sequence designs due to their ability to form liquid crystalline phases in aqueous solution. Each peptide assembles into an antiparallel, homotetrameric coiled-coil bundlemer nanoparticle with overall charges of +24 (SC+6) and +32 (SC+8).^35^ These sequences were chosen because they have different net charges, different amino acid compositions, and different critical liquid crystal concentrations for sake of comparison. To introduce new functionality, both sequences were selectively modified with alloc-protected lysines at the 13th and 19th positions (Table 1), mimicking the site-specific modifications previously made to mixed-charge (MC) 4B+4^32^ sequence that drove nanoporous lattice self-assembly *via* hydrophobic interactions.^40^ The resulting variants, SC+6_2A and SC+8_2A, carry a +5 or +7 charge, respectively (+20 or +28 per bundlemer particle), reflecting the substitution of lysine (positive) and a tyrosine (neutral) residues with alloc-protected lysines.

**Table 1:** Amino acid sequences for single-charge (SC) bundlemer peptides and their alloc-modified variants. Residues at positions a, d, and g in the heptad repeat (abcdefg) form the hydrophobic core. **K̲** represents alloc-protected lysine

Peptide name	a	b	c	d	e	f	g	a	b	c	d	e	f	g	a	b	c	d	e	f	g	a	b	c	d	e	f	g	a	
SC+6	N	T	T	I	Q	K	M	A	T	N	I	R	**K**	M	A	T	S	I	**Y**	K	M	A	T	T	I	Y	K	Q	A	-NH_2_
SC+6_2A	N	T	T	I	Q	K	M	A	T	N	I	R	**K̲**	M	A	T	S	I	**K̲**	K	M	A	T	T	I	Y	K	Q	A	-NH_2_
SC+8	A	R	T	I	Q	T	M	A	S	K	I	R	**K**	M	A	T	S	I	**Y**	K	M	A	T	K	I	Y	K	Q	A	-NH_2_
SC+8_2A	A	R	T	I	Q	T	M	A	S	K	I	R	**K̲**	M	A	T	S	I	**K̲**	K	M	A	T	K	I	Y	K	Q	A	-NH_2_

Circular dichroism (CD) spectroscopy confirmed that the coiled-coil structure was preserved after substitution, showing a characteristic α-helical minima at 208 and 222 nm (Fig. S-2). These results demonstrate that SC bundlemers tolerate site-specific modification without structural disruption. More broadly, they highlight the robustness of the coiled coil assembly and versatility of the computational design framework across both MC and SC sequences.

**Fig. 1:**
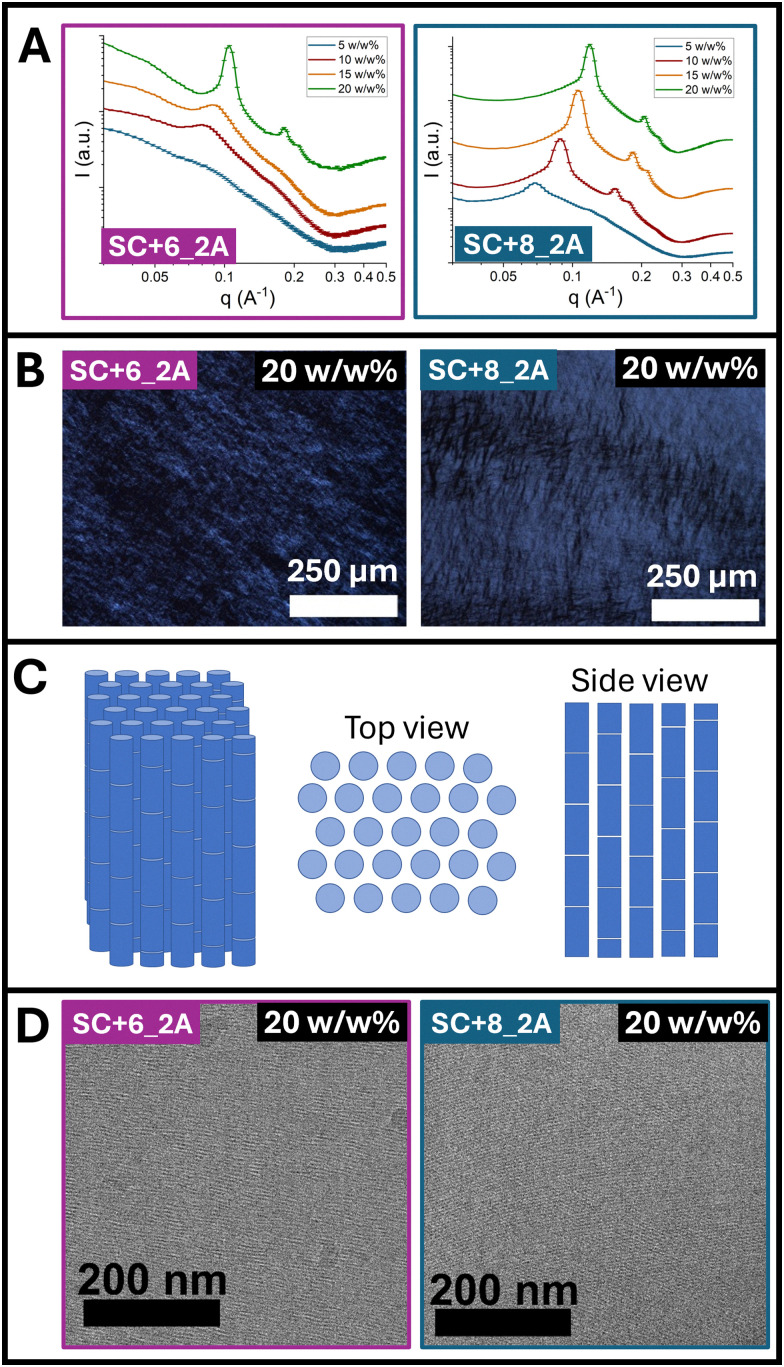
(A) Small-angle X-ray scattering (SAXS) profiles of SC+6_2A (left) and SC+8_2A (right) at 5, 10, 15, and 20 w/w% in Milli-Q water. (B) Polarized optical microscopy (POM) images showing birefringence in SC+6_2A (left) and SC+8_2A (right) at 20 w/w% in Milli-Q water. (C) Schematic model for bundlemer packing in hexagonal column liquid crystals. (D) Cryo-TEM of SC+6_2A (left) and SC+8_2A (right) liquid crystals at 20 w/w% in Milli-Q water.

We evaluated liquid crystal (LC) behavior in water to determine whether alloc modifications to the parent sequence altered assembly. We hypothesized that in salt-free aqueous solution, end-to-end particle stacking and lateral electrostatic repulsion would dominate with alloc side chains non-interacting given the lack of close interparticle contact required for hydrophobic stabilization. Small-angle X-ray scattering (SAXS) data for SC+8_2A and SC+6_2A at 5, 10, 15, and 20 w/w% in Milli-Q water are shown in Fig. 1A. For SC+8_2A, distinct diffraction peaks are present with notable peak positions of q*, 
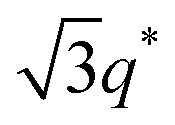
, and 
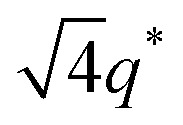
 characteristic of two-dimensional hexagonal packing of stacked bundlemers chains.^42^ The peak positions shifted to higher *q* values with increasing peptide concentration, consistent with tighter packing of hexagonally ordered chains. SC+6_2A exhibited similar behavior, though the hexagonal structure peaks do not appear until 10 w/w% (Fig. 1A). Additionally, the hexagonal diffraction peaks were broader for SC+6_2A, suggesting reduced interchain electrostatic repulsion and weaker long-range ordering compared to SC+8_2A.

LC behavior for both samples was further validated using polarized optical microscopy (POM) (Fig. 1B, with additional, full images for all concentrations in Fig. S-3). SC+8_2A exhibited birefringence at all concentrations examined, thereby exhibiting a critical concentration, defined as the concentration at which birefringence is first observable, below 5 w/w%. In contrast, SC+6_2A showed birefringence only at 10 w/w% and above, indicating its critical concentration is between 5 and 10 w/w%. A schematic model of bundlemer packing for hexagonal columnar liquid crystals is shown in Fig. 1C. Cryo-TEM images of samples at 20 w/w% are shown in Fig. 1D, revealing highly aligned, physically assembled bundlemer chains. Fig. S-5 and S-11 include lower magnification images that highlight the size of the liquid crystal domains and their boundaries. The POM, SAXS, and cryo-TEM results demonstrate that the LC behavior was conserved for both alloc-modified sequences. The SC+6 unmodified parent sequence displayed a similar critical concentration for LC formation, ranging between 5% and 10 w/w%^35^ while the SC+8 unmodified parent sequence displayed a critical concentration between 5 and 10 w/w%,^35^ higher than the observed critical concentration of SC+8_2A below 5 w/w%.

**Fig. 2:**
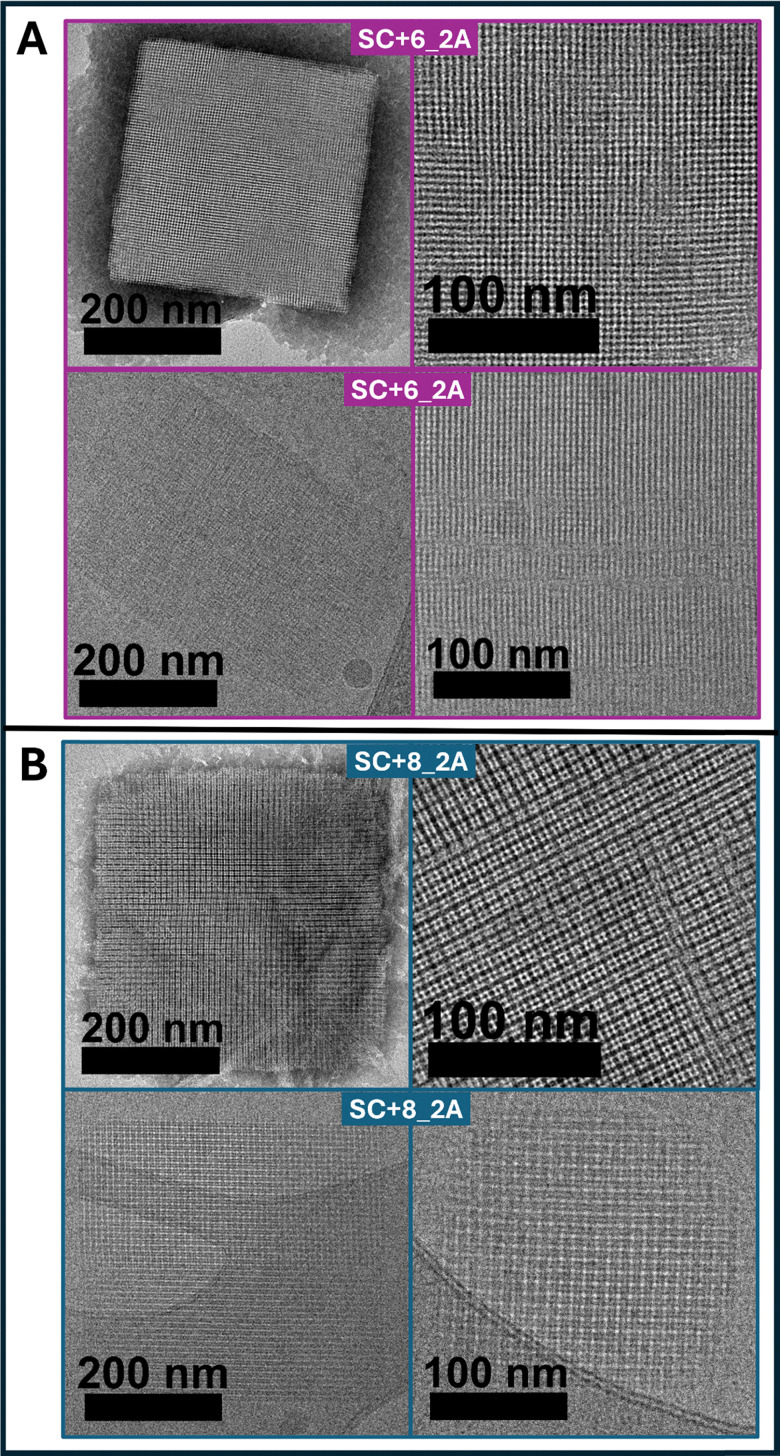
(A) TEM images of SC+6_2A at 5 w/w% in 1 M NaCl. Top: Negative-stain TEM. Bottom: Cryo-TEM. (B) TEM images of SC+8_2A at 5 w/w% in 1 M NaCl. Top: Negative-stain TEM. Bottom: Cryo-TEM.

**Fig. 3:**
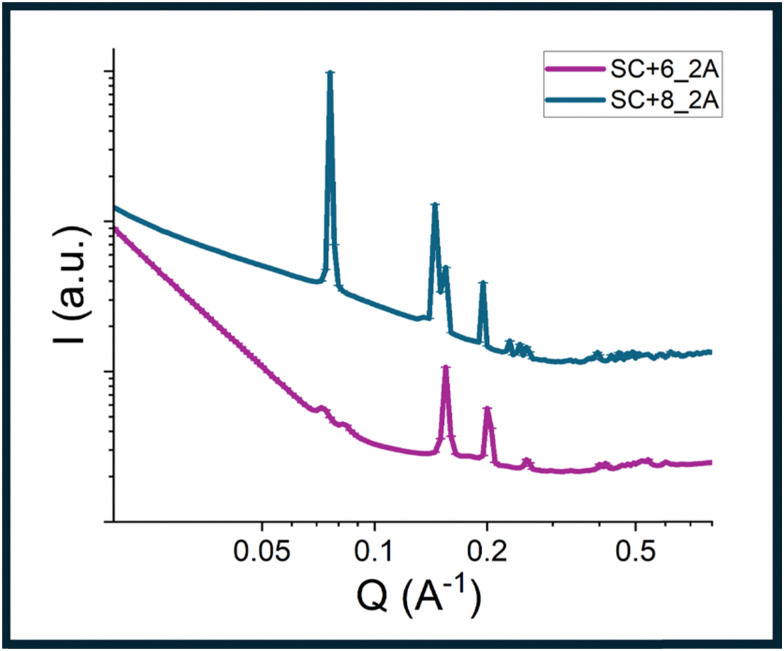
Small-angle X-ray scattering profiles of SC+6_2A at 10 w/w% in 0.5 M NaCl and SC+8_2A at 5 w/w% in 0.5 M NaCl.

In salt-free solution, alloc-modified SC+6_2A and SC+8_2A bundlemers assemble into end-to-end stacked chains that are laterally repulsive, giving rise to hexagonal columnar LC phases. However, by screening electrostatic interactions with added salt, repulsive forces stabilizing a possible hexagonal columnar LC phase will be attenuated, thus allowing hydrophobic alloc side chain interactions to affect interparticle assembly. To test this hypothesis, samples were prepared in 1 M NaCl. Both alloc-modified sequences immediately became turbid on dissolution in the 1 M salt solution. TEM and cryo-TEM (Fig. 2) revealed well-defined nanosheets for both SC+6_2A and SC+8_2A, albeit with distinct lattice projections. SAXS further confirms differences, showing distinct structure factor signature for both sequences (Fig. 3). Thus, despite having the same site-specific alloc modification, SC+6_2A and SC+8_2A assemble into nanoporous lattice structures in solution with subtle but measurable differences in packing. Overall, differences are likely attributable to sequence specific differences in their amino acid composition, particularly within the first two heptads.

**Fig. 4:**
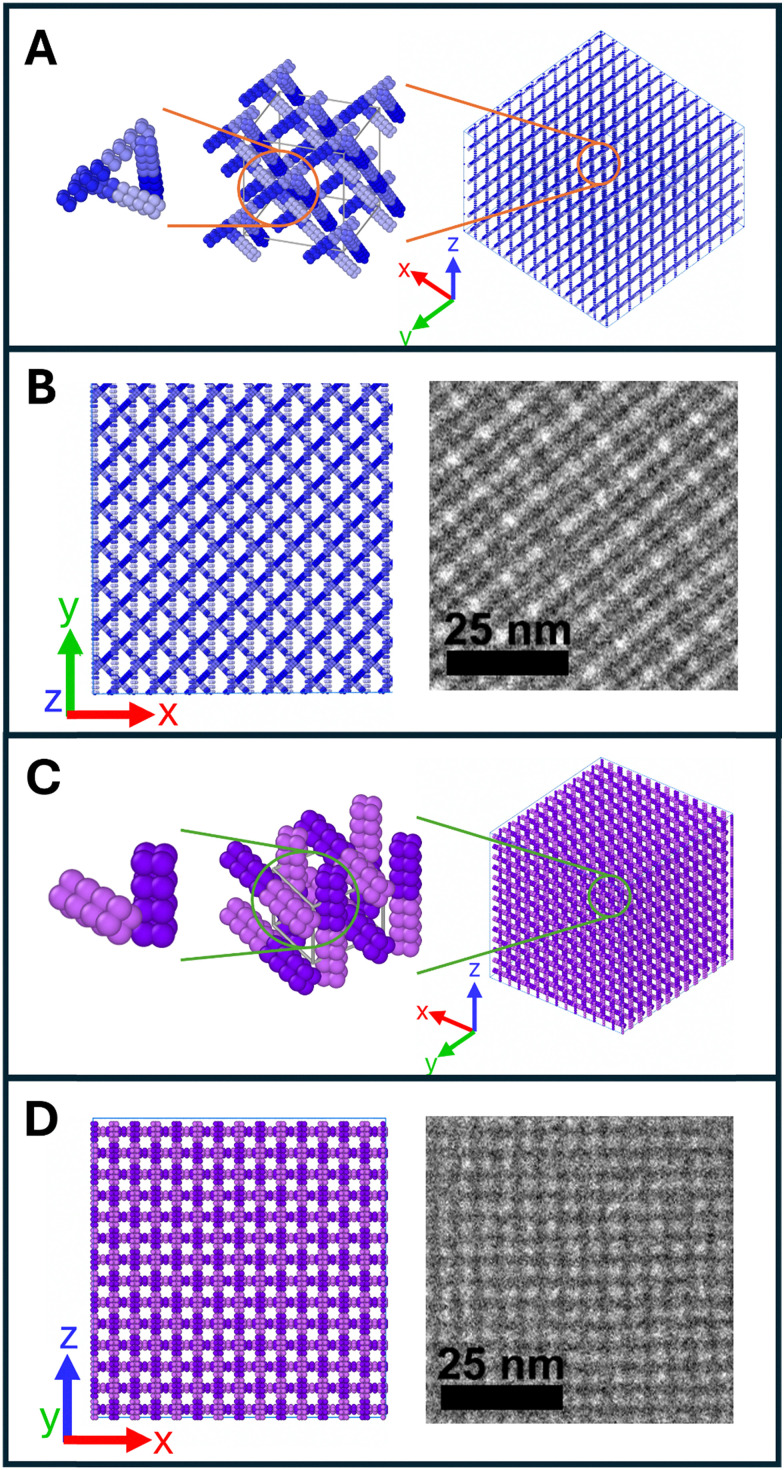
(A) Lattice packing model of SC+8_2A derived from experimental SAXS data (Fig. S-19). (B) Projection of the SC+8_2A lattice model matched to cryo-TEM images, with different hues highlighting individual bundlemers (applied to both SC+6_2A and SC+8_2A). (C) Lattice packing model of SC+6_2A derived from experimental SAXS data (Fig. S-20). (D) Projection of the SC+6_2A lattice model matched to cryo-TEM images.

To study the differences in the two ordered nanostructures, a lattice model was developed for SC+6_2A and SC+8_2A using a machine learning optimization approach to match experimental structure factor data to a coarse-grained bundlemer lattice model.^40^ The resulting model for SC+8_2A is shown in Fig. 4A with projection comparisons for cryoTEM in Fig. 4B. Notably, SC+8_2A exhibited a SAXS signature (Fig. S-17) similar to the previously reported 4B+4_2A lattice, in which a mixed-charged 4B+4 parent sequence carried alloc modifications at the same positions.^40^ This similarity indicates that both sequences assemble into an FCC truss-like lattice with similar bundlemer packing. This observation is particularly noteworthy given the substantial differences in amino acid composition between the two sequences: aside from the shared hydrophobic core (11 residues) and alloc-protected lysines (2 residues at positions 13/19), they share only 4 of 16 surface residues in common. Negative-stain TEM projections of SC+8_2A and 4B+4_2A (Fig. S-18) further highlight the structural similarity in nanostructure between the two lattices. Together, these results demonstrate that once electrostatic interactions are screened, SC+8_2A assembles indistinguishably from the mixed charge 4B+4_2A, underscoring the importance of the alloc placement and hydrophobic interactions in directing lattice formation.

**Fig. 5:**
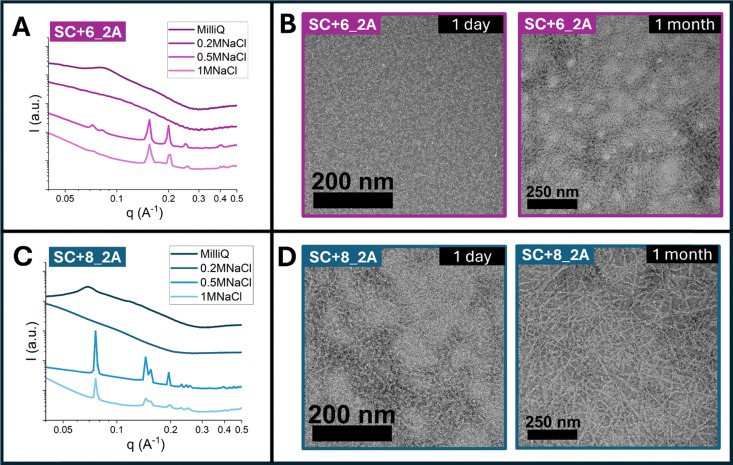
(A) SAXS profiles of SC+6_2A at 10 w/w% (above the critical LC concentration) in Milli-Q water, 0.2 M NaCl, 0.5 M NaCl, and 1 M NaCl. (B) Negative-stain TEM of SC+6_2A at 10 w/w% in 0.2 M NaCl after 1 day (left) and 1 month (right). (C) SAXS profiles of SC+8_2A at 5 w/w% (above the critical LC concentration) in Milli-Q water, 0.2 M NaCl, 0.5 M NaCl, and 1 M NaCl. (D) Negative-stain TEM of SC+8_2A at 5 w/w% in 0.2 M NaCl after 1 day (left) and 1 month (right).

For SC+6_2A, the model was determined by matching several major peaks in the structure factor signature (Fig. 4C). Broad, weak peaks at low *q* were excluded, as they were not consistently observed across salt concentration conditions (Fig. S-16 and Fig. 5A). Only reproducible higher-*q* peaks were used for modeling. The weak low-*q* peaks (*q* = 0.072 Å^−1^, *d* = 8.7 nm; *q* = 0.083 Å^−1^, *d* = 7.6 nm) could be attributed to a lattice sheet thickness or sheet stacking, corresponding to approximately two bundlemers stacked end-to-end or four bundlemers stacked side-by-side. The resulting model indicates that SC+6_2A assembles into lattice sheets with bundlemers aligned end-to-end within a layer and neighboring layers oriented perpendicular to one another, yielding a square lattice morphology. As observed in other bundlemer lattice models, the assembly reflects coupled end-to-end stacking and side-to-side hydrophobic alloc interactions. Experimental cryo-TEM images matched the computational model derived from structure factor signature in SAXS (Fig. 4D), showing a square-like lattice with clear four-fold projections.

Given that electrostatic interactions, coupled with end-to-end particle stacking, drive LC self-assembly, salt screening plays a vital role in enabling hydrophobic alloc interactions and consequent lattice formation. To probe this effect and identify the optimal salt concentration for lattice formation, sequences were dissolved directly in NaCl solutions at 0, 0.2, 0.5, and 1 M. The corresponding Debye screening lengths from the added NaCl are approximately 0.7 nm (0.2 M), 0.4 nm (0.5 M), and 0.3 nm (1 M). By comparing assembled nanostructures across these conditions, we aimed to determine the screening length at which hydrophobic side-chain interactions overcome electrostatic repulsion to reorganize bundlemers into lattice assemblies. Both SC+6_2A and SC+8_2A were studied above their critical LC concentration (10 w/w% and 5 w/w%, respectively), and nanostructure was evaluated by SAXS. Fig. 5A and C show SAXS data for SC+6_2A and SC+8_2A. In pure water (0 M NaCl), both sequences exhibited LC behavior with hexagonal packing, consistent with the broad diffraction peaks and observed previously in Fig. 1A. At 0.2 M NaCl, electrostatic screening disrupted liquid crystallinity, but no distinct lattice diffraction peaks were observed. At 0.5 M NaCl, distinct diffraction peaks were observed, indicating lattice formation; thus, the LC-to-lattice transition occurs between screening lengths of 0.7 nm and 0.4 nm, requiring close bundlemer proximity for hydrophobic stabilization. At 1 M NaCl, lattice peaks persisted but were weaker, suggesting weaker long-range order in the lattice assembly, likely due to the formation of many smaller lattice particles locally from the high salt concentration. Overall, these data provide a window for the optimized salt concentration for lattice formation centered around 0.5 M NaCl, while intermediate salt concentrations (*e.g.*, 0.2 M) effectively disrupt both phases, leaving individual bundlemers and shorter bundlemer chains dispersed without ordered structure.

To assess whether such disordered states could evolve over time, the 0.2 M NaCl solutions were aged (at room temperature) and then imaged by TEM (Fig. 5B and D). After one day, both sequences exhibit mostly nonspecific aggregation with a small number of nanofibrils forming/growing through end-to-end stacking. After one month, fibril content increased, but these fibrils did not organize into LC or lattice phases, consistent with electrostatic screening preventing side-to-side interactions as well as disrupting the required end-to-end stacking for LC formation. Together, these results demonstrate the polymorphic nature of the designed nanoparticles: depending on salt concentration, the same sequences can assemble into LC phases, nanoporous lattices, or disordered nanofibrils. This tunability demonstrates how simple control over electrostatic screening can dictate fundamentally different self-assembled states.

Direct solubilization of bundlemer particles into either pure water or concentrated salt demonstrated that liquid crystals or crystal lattices could be selectively formed, confirming the hypothesized self-assembly behavior of alloc-modified SC bundlemers. To further probe the responsiveness of these assemblies, we investigated whether transitions between the LC and lattice ordered phases could be induced by varying salt concentration. This transition proved challenging due to both the high stability and viscosity of the LC phases and the distinct particle orientations required for the two structures: LC domains form parallel, end-to-end hexagonal columnar chains,^35^ while nanoporous lattices adopt a truss-like structure in which bundlemer chains meet at sharp, perpendicular angles. Therefore, the interparticle interactions stabilizing the LC state must be weakened or broken, and bundlemer orientation must be significantly reorganized for lattice formation.

**Fig. 6:**
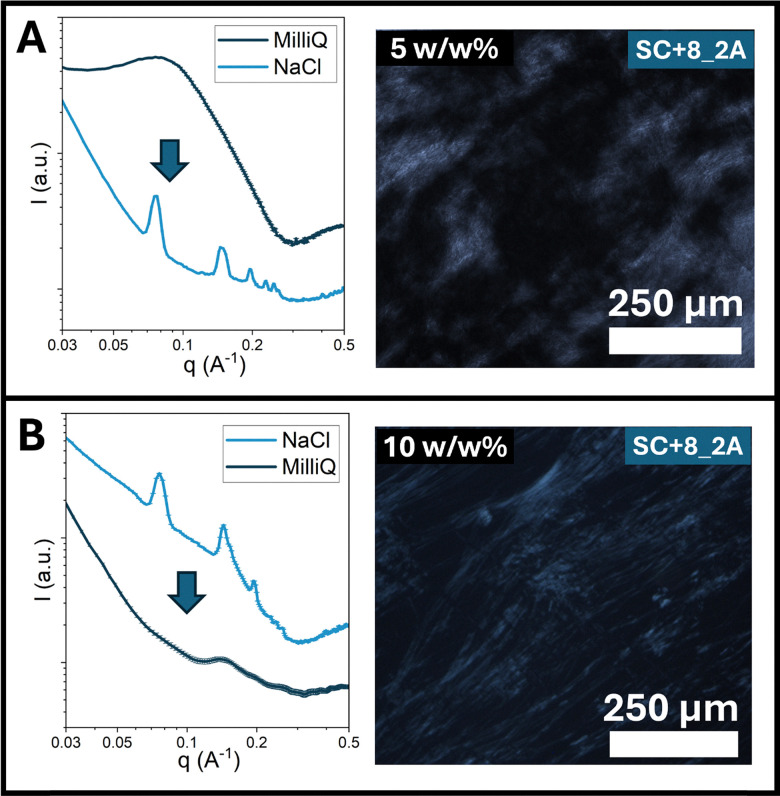
(A) Order-to-order of SC+8_2A prepared at 5 w/w% in Milli-Q water, forming nematic liquid crystals confirmed by SAXS (left) and POM (right). After salt addition and sonication, lattice particle formation was confirmed by SAXS (left). (B) Order-to-order transition of SC+8_2A prepared at 10 w/w% in 0.5 M NaCl, forming lattice particles confirmed by SAXS (left). Following solvent exchange to remove salt, nematic liquid crystal formation was confirmed by SAXS (left) and POM (right).

We first examined the LC to lattice transition. SC+8_2A, chosen for the similarity of the lattice it formed to previously studied mixed-charge lattices, was solubilized in Milli-Q water, producing LC phases, and subsequently dialyzed against concentrated NaCl. Dialysis yielded no conversion of the LC phase to lattices; instead, samples resembled those observed previously at 0.2 M NaCl, containing short nanofibrils formed by partial disruption of side-to-side interactions in the liquid crystal chains. These results suggest that the diffusion of salt within the LC phase alone was insufficient to drive reorganization, as partial charge screening may reinforce parallel chain orientation by promoting local hydrophobic contacts within the LC state. In contrast, quenching the LC solutions with concentrated NaCl followed by sonication proved effective in lattice formation. SC+8_2A at a 5 w/w% formed nematic LCs, as confirmed by SAXS and POM (Fig. 6A). Upon rapid salt addition, mixing, and sonication (see Methods), SAXS revealed the emergence of lattice peaks, and birefringence decreased, consistent with the partial conversion to lattice particles. Sonication was critical for disrupting interparticle interactions in the LC state and enabling reorganization into the nanoporous lattice state.

Driving the reverse transition, from the lattice state to the liquid crystal phase, similarly proved challenging. Dialysis of SC+8_2A prepared in 0.5 M NaCl did not restore LC phases, likely due to the stability of the already formed, hydrophobically stabilized lattice and, potentially, residual salt that suppresses LC formation. To overcome this, we employed solvent exchange with centrifugal filtration. SC+8_2A prepared at 10 w/w% in 0.5 M NaCl was confirmed to form lattices by SAXS (Fig. 6B). These samples were diluted with Milli-Q water, washed by centrifugal filtration to remove salt while retaining peptides, then reconcentrated and sonicated to minimize nonspecific aggregation. After salt removal, the SAXS showed the disappearance of lattice peaks, and POM showed new regions of strong birefringence, consistent with the formation of a nematic liquid crystal phase *via* this method. Together, these results highlight the robustness of both the LC and nanoporous lattice assemblies and demonstrate that significant physical agitation or solvent processing is required to overcome the barriers between them for order-order transitions to occur.

## Conclusions

We have demonstrated that through intentional design and amino acid substitution, we can create peptide nanoparticles that are capable of adopting two distinct ordered phases simply by altering solution conditions to manipulate electrostatic screening. Single-charge (SC) bundlemers, selectively modified with hydrophobic alloc side chains, exhibited polymorphic behavior, assembling into liquid crystals in pure water and nanoporous lattices in concentrated salt. Notably, the bundlemers retain the same folded coiled coil state in both phases, but modulation of surface interactions directs their unique interparticle configurations. These results demonstrate both the robustness of the LC phase in SC sequences and the ability to introduce new functionality without disrupting their native assembly. Under electrostatic screening conditions, the alloc-driven hydrophobic interaction dominated, producing nanoporous lattices across both SC and the previously studied mixed-charge bundlemer sequences^40^ despite significant differences in amino acid composition. This convergence highlights the importance of functional group placement on the bundlemer periphery in dictating targeted self-assembly. The functional design of these peptide particles establishes a versatile platform for tunable nanostructures, enabling bottom-up fabrication of peptide-based materials. A single molecule capable of adopting two distinct ordered phases offers new opportunities in templating, sensing, or smart material applications. Moreover, the ability to introduce site-specific modifications at the individual amino acid resolution provides protein-like precision in programming interparticle interactions and nanostructure formation.

## Methods

### Peptide synthesis

Peptides were synthesized *via* microwave-assisted solid-phase peptide synthesis (SPPS) using a CEM Liberty Blue system. Rink amide resin (loading varying from 0.3–0.6 mmol g^−1^) was used as the solid support. Fmoc-protected amino acids were prepared at 0.2 M in dimethylformamide (DMF), and coupling agents oxyma and diisopropylcarbodiimide were prepared at 1 M. Fmoc deprotection was achieved with 20 v/v% piperdine in DMF. Coupling reactions were preformed at 90 °C for 4 min.

### Peptide cleavage

Following synthesis, resin was sequentially triple-washed with DMF, methanol (MeOH), and dichloromethane (DCM). Resin was dried under nitrogen gas for 15 min. Peptides were cleaved from the resin with a cocktail of 90 v/v% trifluoroacetic acid (TFA), 5 v/v% triisopropylsilane (TIPS), and 5 v/v% Milli-Q water, supplemented with phenol and dithiothreitol (DTT) (50 mg mL^−1^). For a 0.25 mmol scale synthesis, 20 mL of cleavage cocktail was used and the resin was shaken using a wrist-action shaker for 2 h. After cleavage, crude peptide was precipitated by dropwise addition into chilled diethyl ether and pelleted by centrifugation (4000 rpm, 5 min). The supernatant was discarded and the pellet was washed twice with chilled diethyl ether and dried.

### Peptide purification

Dried pellets were dissolved in Milli-Q water and purified *via* reverse-phase high-performance liquid chromatography (HPLC) (Waters 2535 Quaternary Gradient Module) on a C18 column, using water (0.1 v/v% TFA) and acetonitrile (0.1 v/v% TFA) as mobile phases with a 1% min^−1^ gradient (from 20% to 60% acetonitrile). HPLC fractions were analyzed by ultraperformance liquid chromatography-electrospray ionization-tandem mass spectrometry (UPLC/ESI-MS). Pure fractions were frozen in liquid nitrogen and lyophilized for 3 days. Lyophilized peptides were stored at −20 °C.

### Circular dichroism (CD) spectroscopy

CD spectra were acquired on a JASCO 1500 spectrometer with peptide solutions at 0.1 mM in Milli-Q water using 1 mm quartz cuvettes at 20 °C. Spectra were collected from 250 to 190 nm, averaging three accumulations.

### Polarized optical microscopy (POM)

Samples were prepared at 5 to 20 w/w% in Milli-Q aged at room temperature for 3 days before imaging. 5 µL of each sample was pipetted onto a clean glass microscope slide and A glass coverslip was placed on the sample. Imaging was conducted on a Nikon Eclipse LV100 POL microscope equipped with a Nikon DS-Ri1 camera. Images were captured using NIS-Elements software and processed using Adobe Photoshop.

### Transmission electron microscopy (TEM)

TEM grids were purchased from Electron Microscopy Sciences. Ultrathin carbon with a mesh size of 200 or 400 were used. Grids were glow-discharged for 30 seconds using Pelco easiGlowTM glow discharge cleaning system. Samples were diluted to approximately 1–5 mM for each grid without mixing and deposited directly on the grids. 5 µL of peptide solution was pipetted and allowed to sit for 60 seconds before being wicked away using a KimWipe. 5 µL of 2 w/v% phosphotungstic acid (PTA) stain was added and allowed to sit for 50 seconds before being wicked away. Grids were left to dry in ambient conditions for at least 10 minutes before imaging. Samples were imaged on an FEI TALOS F200C TEM using an accelerating voltage of 200 kV. Image processing was conducted in Adobe Photoshop.

### Cryogenic-transmission electron microscopy (cryo-TEM)

Lacey carbon type C TEM grids were purchased from Ted Pella, Inc. Grids were glow discharged for 30 seconds using Pelco easiGlowTM glow discharge cleaning system. No dilutions were conducted before sample preparation. Samples were prepared using a Vitrobot with a 100% humidity chamber. Samples were imaged on a FEI TALOS F200C TEM using an accelerating voltage of 200 kV. Image processing was conducted in Adobe Photoshop.

### Small angle X-ray scattering (SAXS)

Quartz capillaries with a thickness of 0.01 mm and an outer diameter of 1.5 mm were used (Charles Supper Company). Sample concentration varied for experiments. Experiments were conducted using two different sources: NSLS-II 16-ID (LiX) at Brookhaven National Laboratory (BNL) and Xeuss Xenocs 2.0. Data from Brookhaven National Laboratory was obtained at NSLS-II 16-ID (LiX) beamline with a beam energy of 15.1 keV (0.82 Å). Exposure time was 0.5 s for each sample. Data obtained on a Xeuss Xenocs 2.0 used SAXS1 and standard exposure with exposure times ranging from 2–6 hours per sample. The beam energy was 8 keV (1.55 Å). All intensity data are plotted relative to the scattering wave vector *q* = 4π[sin(*θ*/2)]/*λ* where *θ* is the scattering angle measured in the transmission SAXS geometry and *λ* is the X-ray wavelength.

### Debye length calculations

The Debye length (*λ*_D_, nm) for NaCl solutions was calculated as:
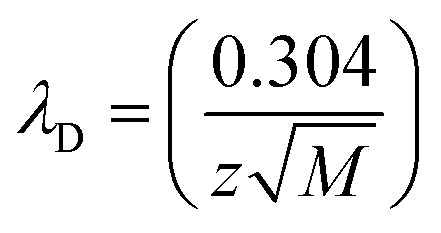
where *M* is the salt molarity (mol L^−1^) and *z* is the ion valency.^43^

### Liquid crystal to lattice transition

Solutions were prepared in Milli-Q water. LC formation was confirmed *via* POM. Transition to the lattice state was induced by adding a 5 M NaCl to a final concentration of 1 M, followed by manual mixing and bath sonication for 2 h day^−1^ for several days. Lattice formation was confirmed *via* SAXS.

### Lattice-to-liquid crystal transition

Solutions (10 w/w%) were prepared in 0.5 M NaCl and confirmed to form lattices using SAXS. Transition was induced *via* a solvent exchange: 50 µL of peptide solution was loaded into a centrifugal concentrator (Amicon® Ultra Centrifugal Filter, 0.5 mL, regenerated cellulose membrane, 3 kDa MWCO), diluted with 200 µL Milli-Q water, placed in a microcentrifuge and spun at 14 100 rcf for 30 min. This wash step was repeated three times. To achieve slow evaporation, samples were placed on a thermomixer set at 45 °C and 500 rpms. Samples were heated for 3–4 hours to achieve approximately 50% solution evaporation. After evaporation, samples were placed in a bath sonicator heated at 40 °C for 3 hours. Samples were then aged at room temperature for 3 days before POM imaging.

### Lattice structure modelling

Lattice structures were determined following published protocols using coarse-grained (CG) modeling, structure factor calculations, and machine learning Bayesian optimization techniques.^35,40^ For SC + 6_2A, the unit cell was modeled two orthogonally oriented bundlemers, with interparticle distances (*x*_d_, *y*_d_, *z*_d_) and unit cell dimensions (*x*_u_, *y*_u_, *z*_u_) optimized against SAXS peaks. Here, the 4 major peaks from 3rd to 6th in SAXS were used for the optimization. The lowest mean absolute error (0.02 nm) gave parameters: *x*_d_ = 0.28, *y*_d_ = 2.49, *z*_d_ = 0.37, *x*_u_ = 4.0, *y*_u_ = 4.98, *z*_u_ = 3.98 nm. The detailed information about the modeling is provided in previous work.^35^ For SC + 8_2A, the assembly was modeled as an FCC truss lattice comprising three end-to-end bundlemer pairs, as shown in Fig. 4. Initial lattice parameters (pair distance, *L*, and Cartesian coordinates of the bundlemer pairs) were obtained by fixing one pair along the *y* axis and generated the other two pairs to match the relative strut orientations within the FCC truss lattice. Optimization was performed against the five major peaks in SAXS, yielding a lowest mean absolute error of 0.07 nm with parameters: *L* = 4.98, *x*_1_ = −3.65, *y*_1_ = −0.42, *z*_1_ = 2.29, *x*_2_ = −2.47, *y*_2_ = 1.48, *z*_2_ = 3.30, *x*_3_ = −3.36, *y*_3_ = 2.81, *z*_3_ = −1.94. Detailed protocols are reported in previous work.^40^

## Conflicts of interest

There are no conflicts to declare.

## Supplementary Material

SM-022-D5SM00972C-s001

## Data Availability

Additional data supporting this article is included in the supplementary information. Supplementary information: contains mass spectroscopy, CD spectroscopy, supplemental TEM, SAXS, and lattice models. See DOI: https://doi.org/10.1039/d5sm00972c.
