# Supplementary material for: Designed coiled-coil peptide nanoparticles with tunable self-assembly: distinct ordered nanostructures via nonnatural side chain modification and electrostatic screening
Source: Soft Matter. 2025 Dec 1;22(2):428–37. doi: 10.1039/d5sm00972c (PMC12694727; doi:10.1039/d5sm00972c)
Supplement: SM-022-D5SM00972C-s001 [file SM-022-D5SM00972C-s001.pdf]

Supplementary Information (SI) for Soft Matter.
This journal is © The Royal Society of Chemistry 2025

Supporting Information

TITLE: Designed coiled-coil peptide nanoparticles with tunable self-assembly: distinct ordered nanostructures via nonnatural side chain modification and electrostatic screening

AUTHORS: Amanda L. McCahill[1], Tianren Zhang[1,3], Yi Shi[1], Jacob Schwartz[1], Christopher J. Kloxin[1,2], Jeffery G. Saven[3], Darrin J. Pochan[1]

[1]Department of Materials Science and Engineering, University of Delaware, Newark, DE 19716
[2]Department of Chemical and Biomolecular Engineering, University of Delaware, Newark, DE 19716
[3]Department of Chemistry, University of Pennsylvania, Philadelphia PA, 19104

Corresponding Author: Darrin J. Pochan (pochan@udel.edu)

Table of Contents

I. Liquid Chromatography – Mass Spectrometry (LC-MS)
II. Circular Dichroism (CD) Spectroscopy
III. Polarized Optical Microscopy (POM)
IV. Supplemental Transmission Electron Microscopy (TEM)
V. Supplemental Small Angle X-ray Scattering (SAXS)
VI. Data Comparisons
VII. Lattice Packing Models

I. Liquid Chromatography – Mass Spectrometry (LC-MS)

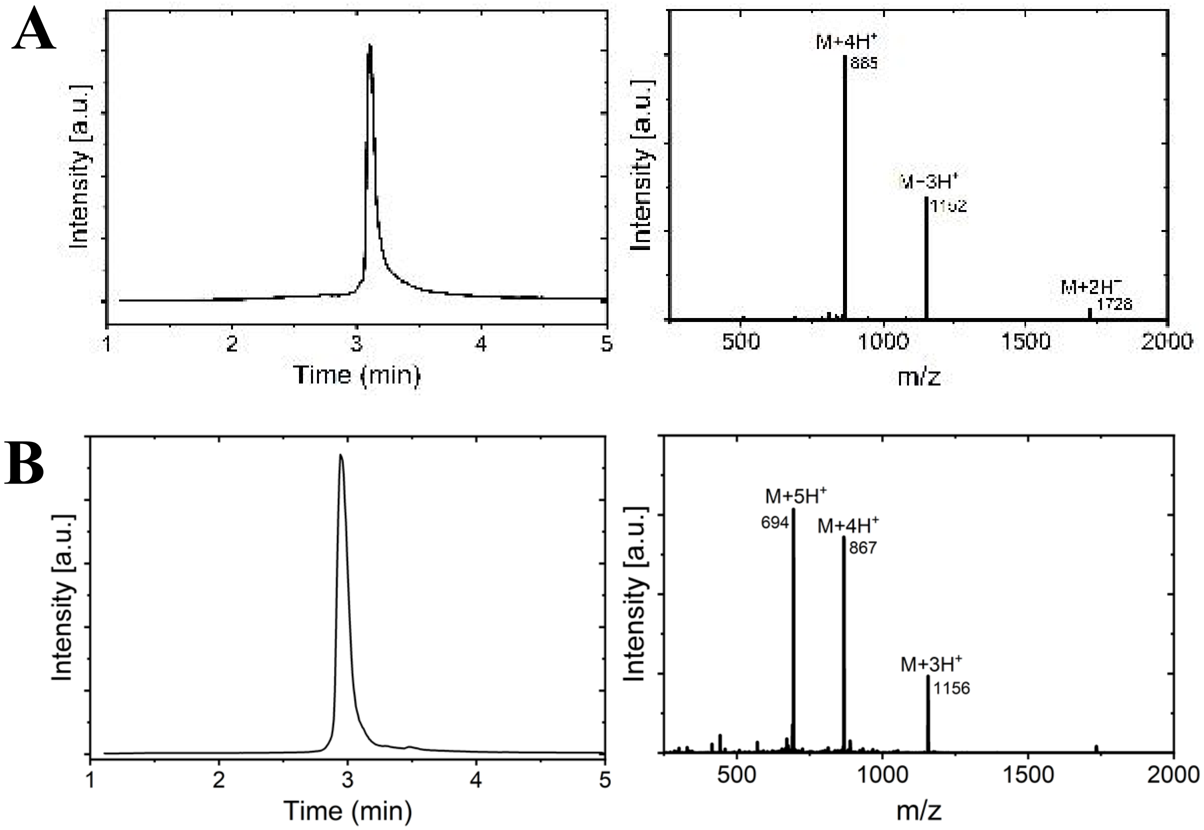

Figure S-1: UPLC-ESI-MS/MS data for (A) SC+6_2A (3454 g/mol) and (B) SC+8_2A (3466 g/mol). Includes the full UPLC trace (left) and the ESI trace (right) for the major peak.

II. Circular Dichroism (CD) Spectroscopy

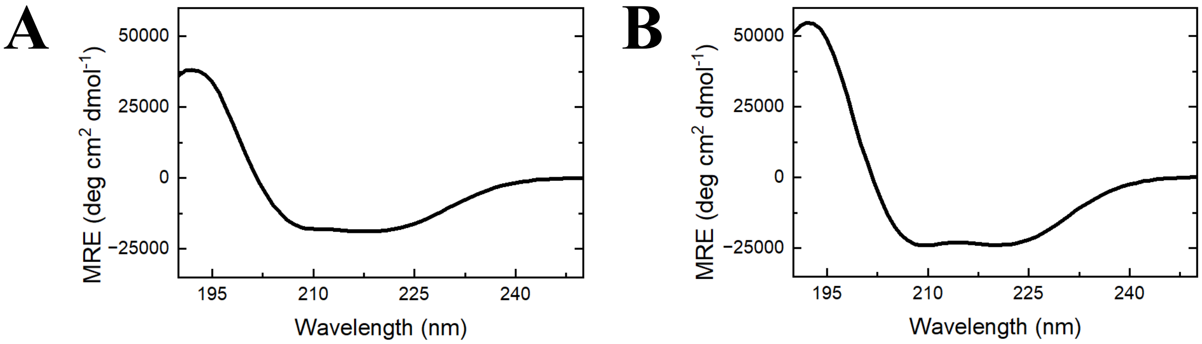

Figure S-2: CD Spectroscopy data for (A) SC+6_2A and (B) SC+8_2A at 0.1 mM concentration in MilliQ. Data taken at 20 °C.

III. Polarized Optical Microscopy (POM)

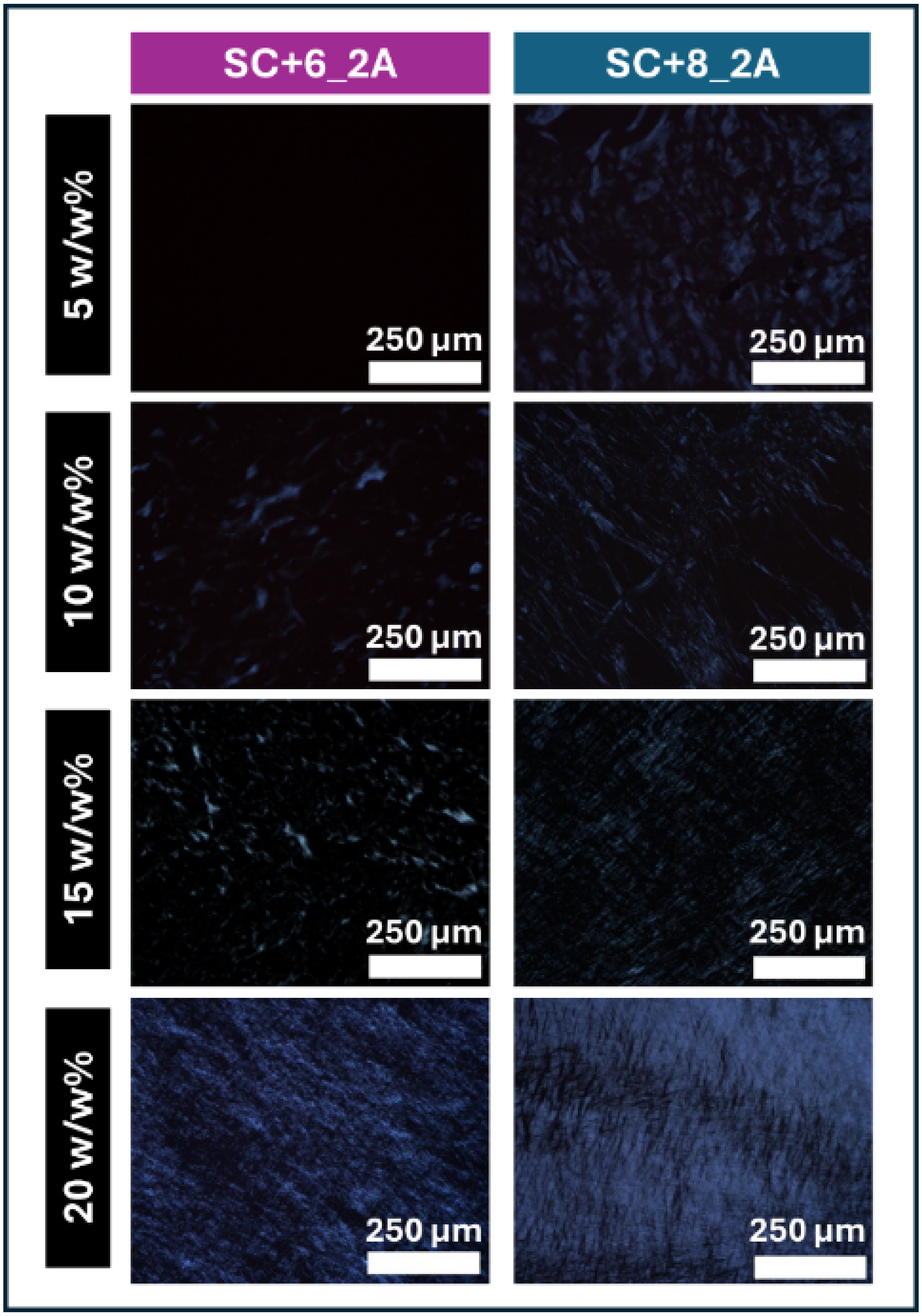

Figure S-3: Polarized optical microscopy (POM) results for SC+6_2A (left) and SC+8_2A (right) at 5, 10, 15, and 20 w/w% in MilliQ. Scale bar for all images is 250 µm.

IV. Supplemental Transmission Electron Microscopy (TEM)

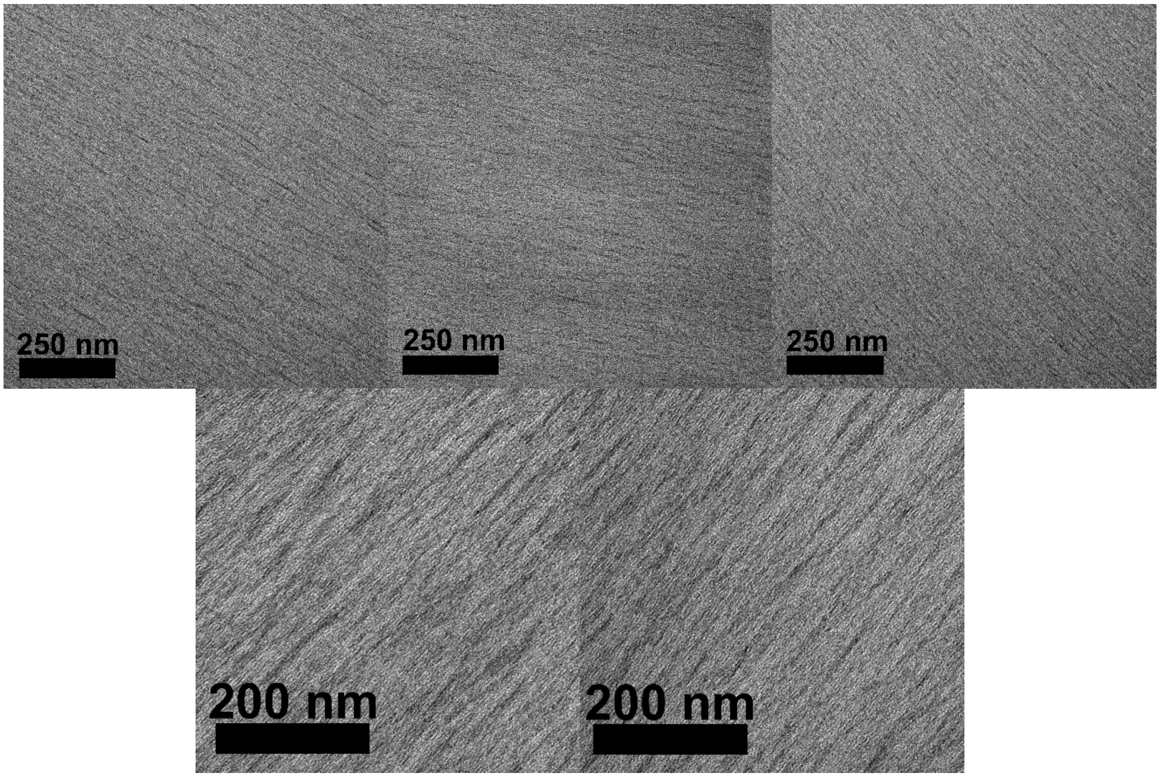

Figure S-4: Negative Stain TEM of SC+8_2A prepped at 20w/w% in MilliQ (diluted on grid to minimize dilution effects).

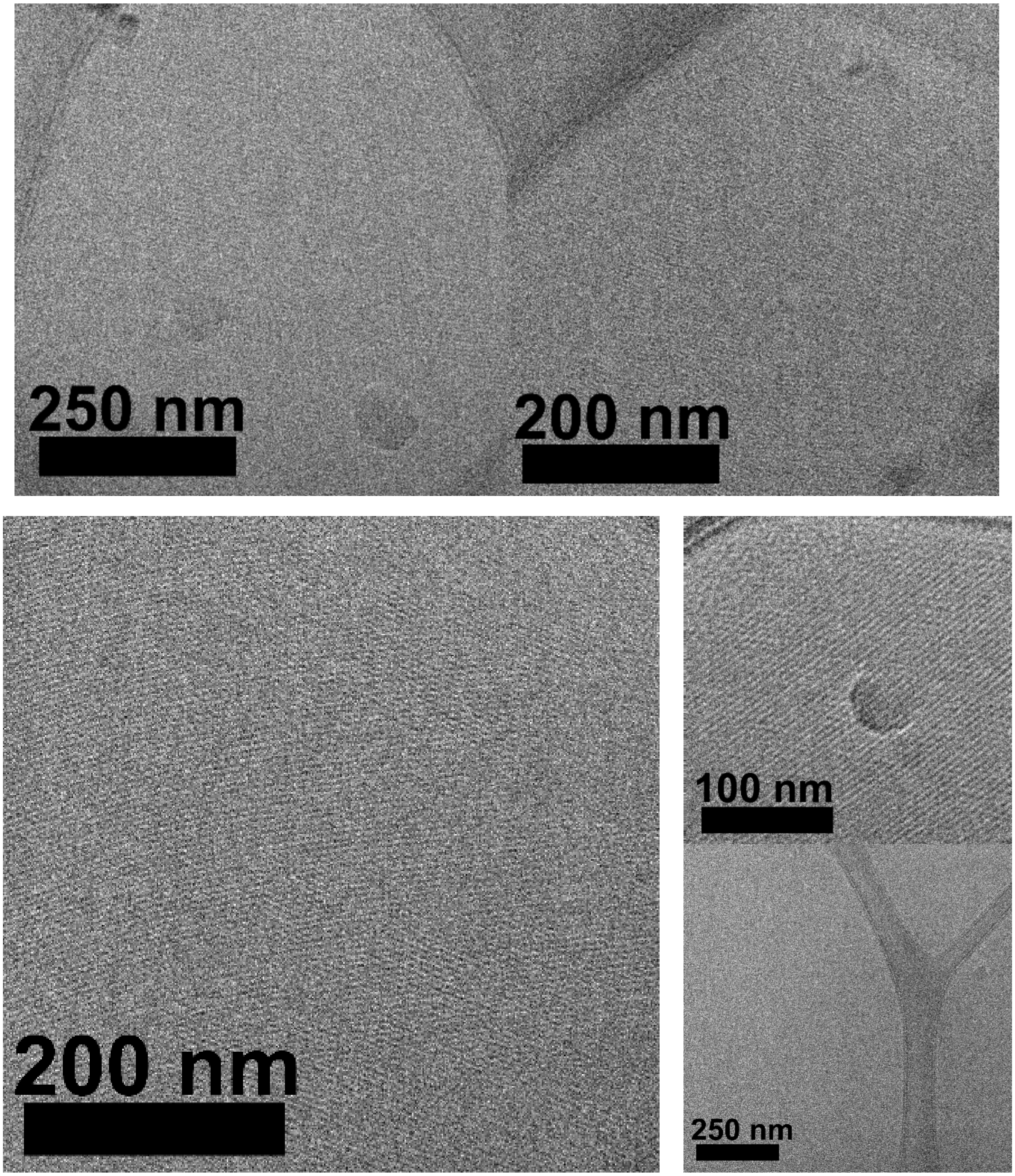

Figure S-5: CryoTEM of SC+8_2A prepped at 20w/w% in MilliQ.

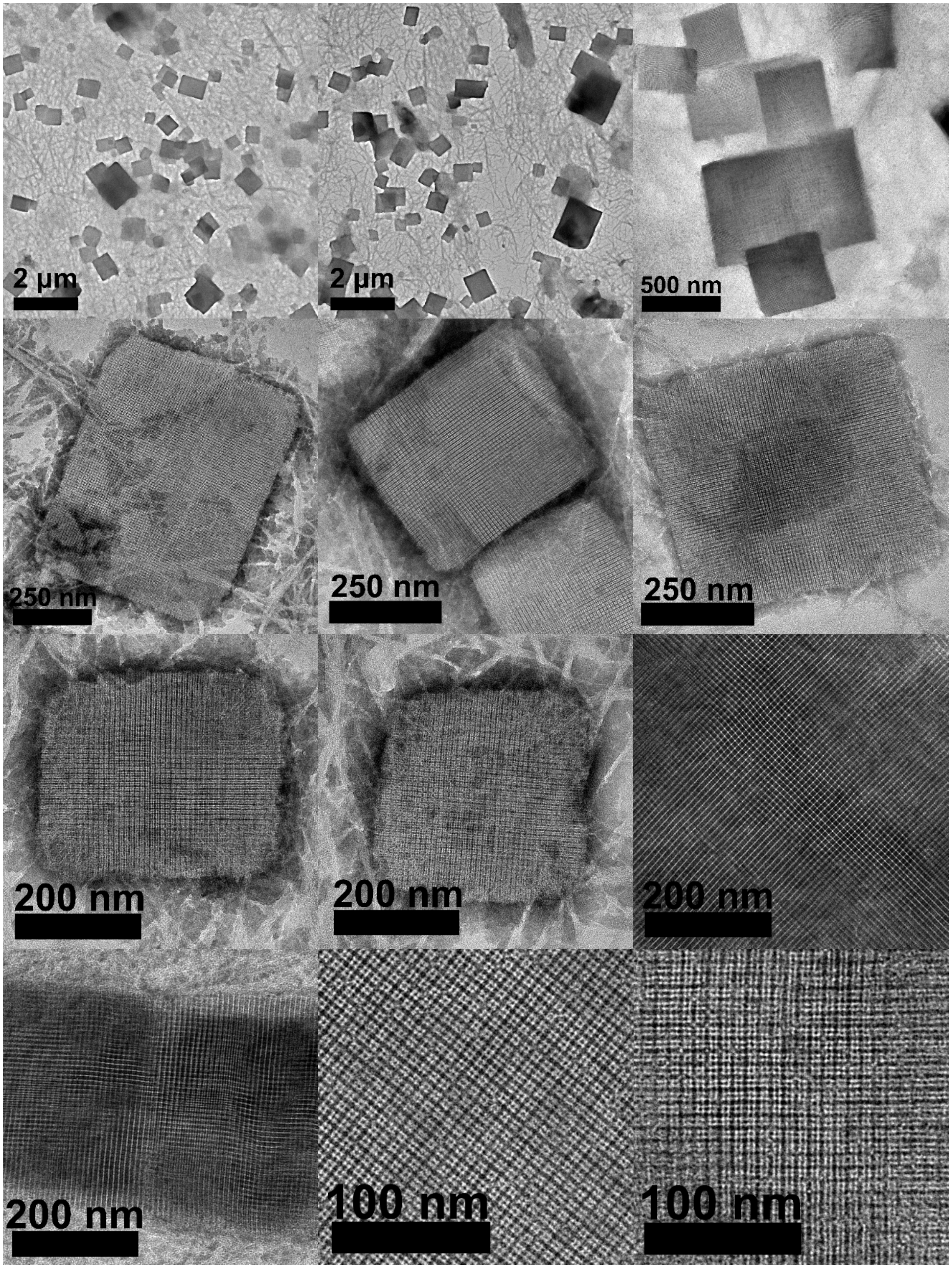

Figure S-6: Negative Stain TEM of SC+8_2A at 5w/w% in 1M NaCl

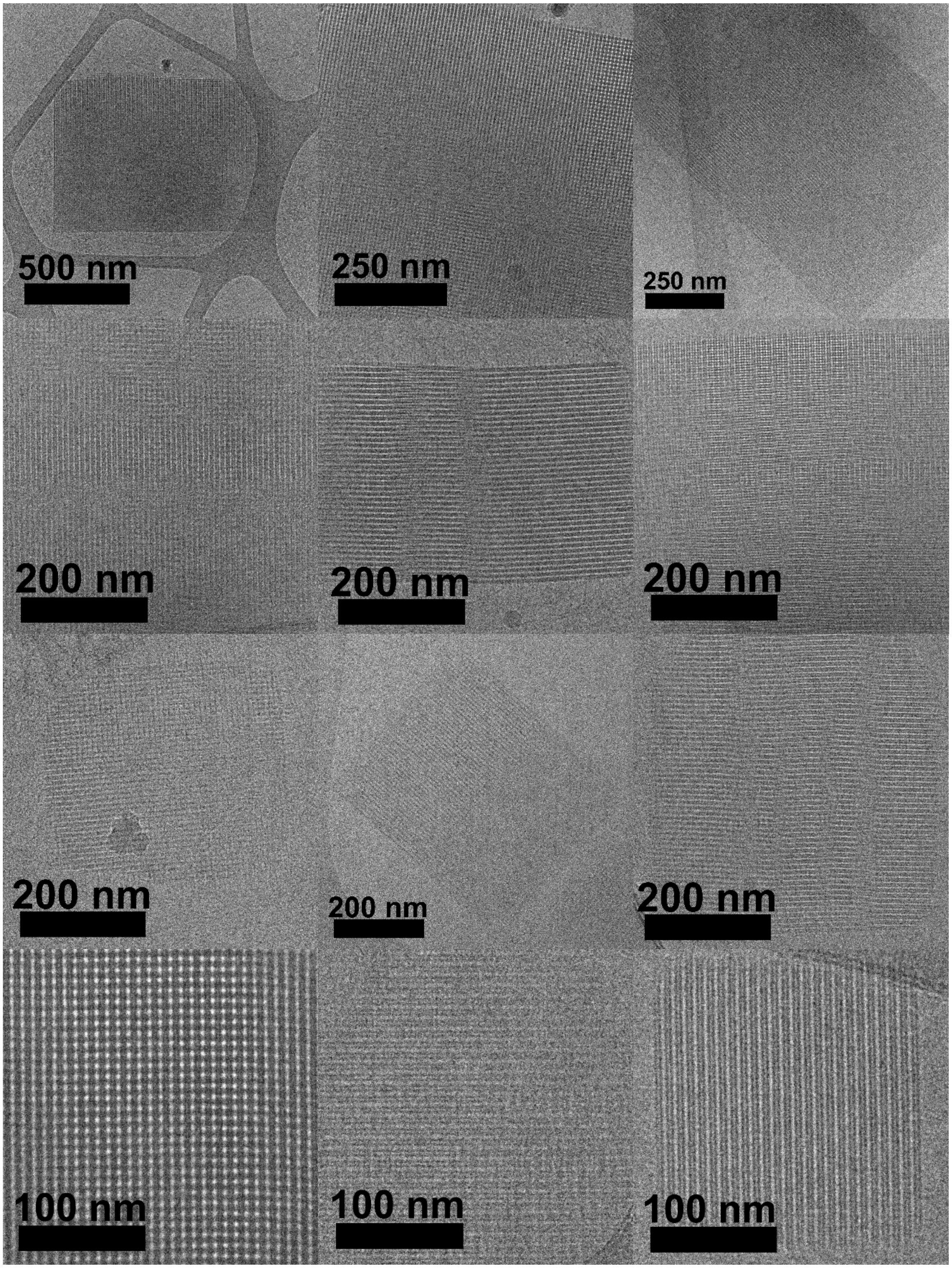

Figure S-7: Cryo TEM of SC+8_2A at 5w/w% in 1M NaCl

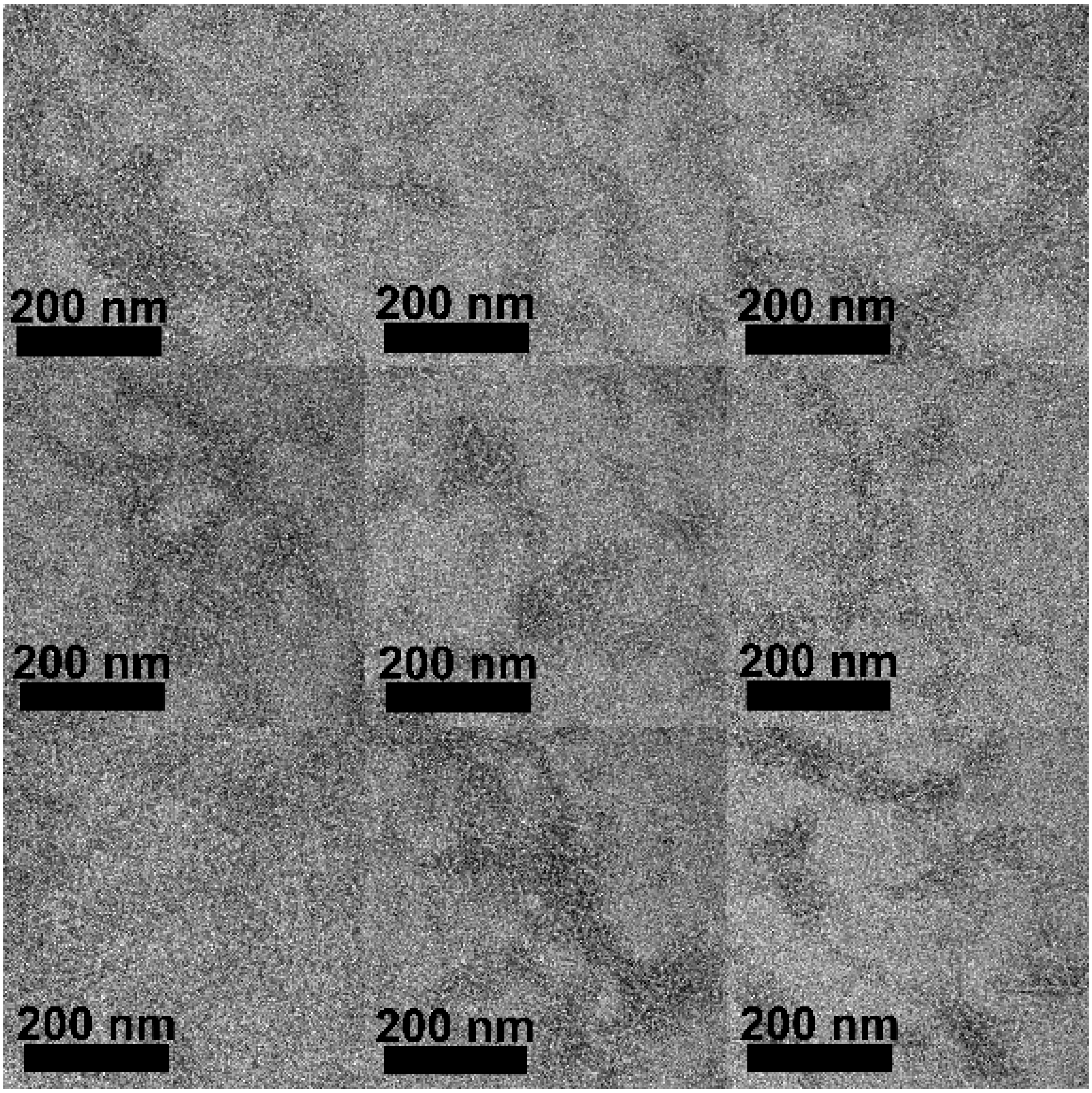

Figure S-8: Negative Stain TEM of SC+8_2A at 5w/w% in 0.2M NaCl aged 1 day

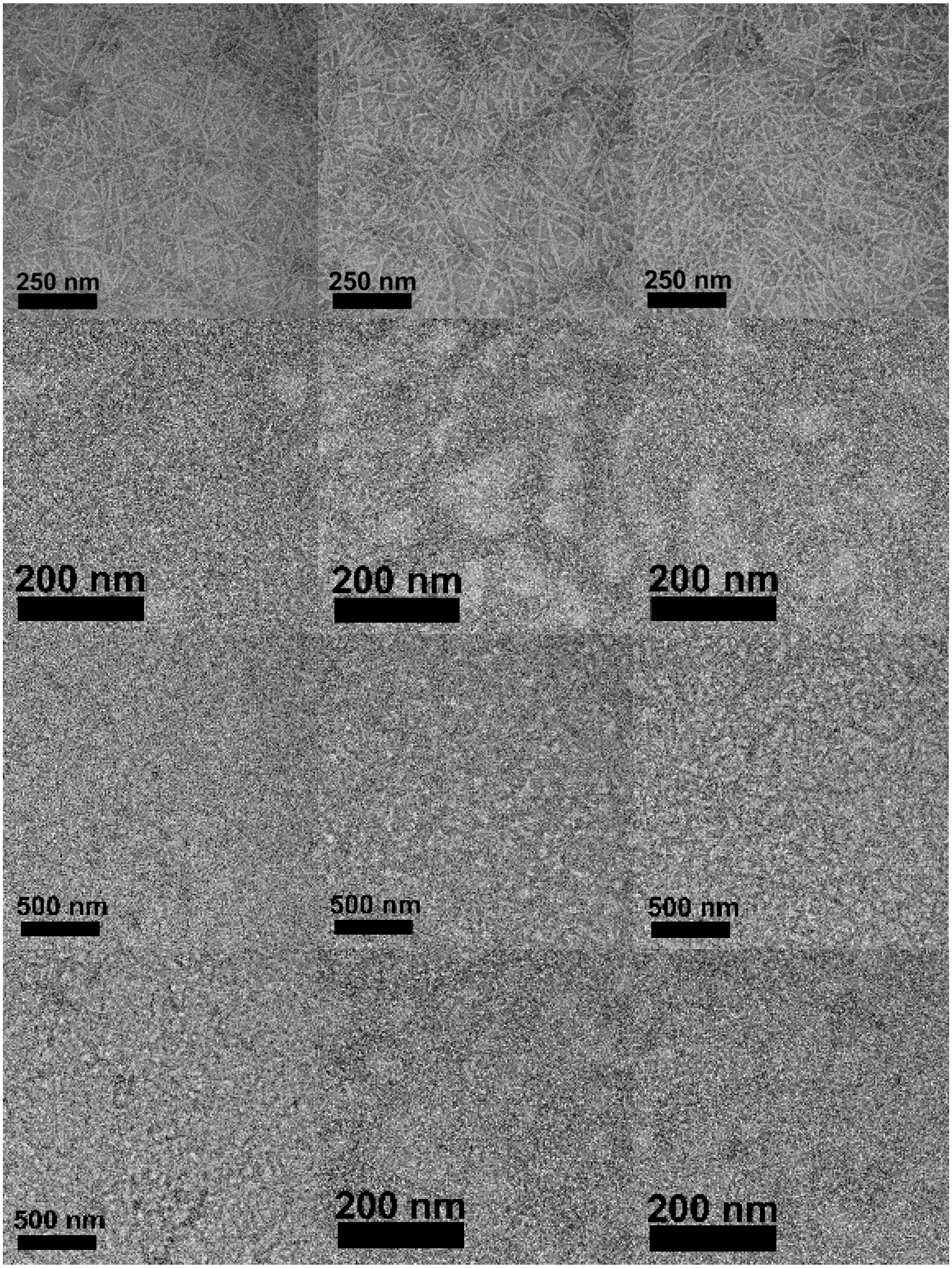

Figure S-9: Negative Stain TEM of SC+8_2A at 5w/w% in 0.2M NaCl aged 1 month

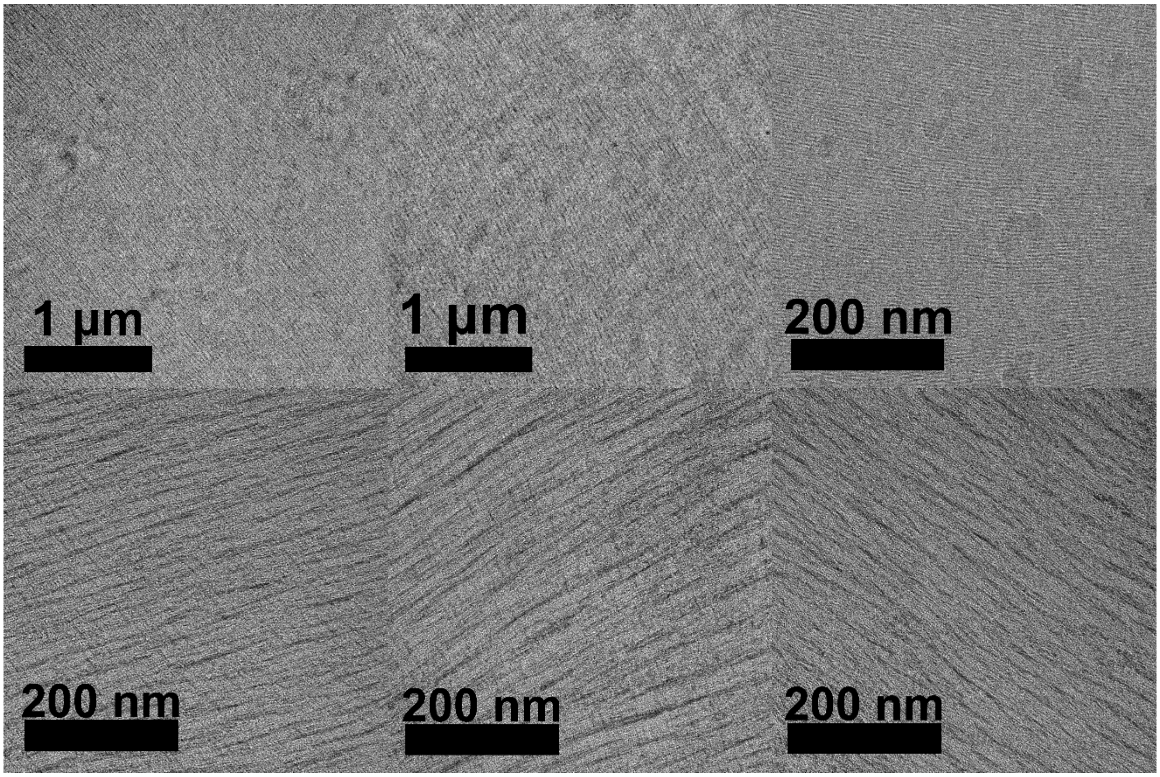

Figure S-10: Negative Stain TEM of SC+6_2A prepped at 10w/w% in MilliQ (diluted on grid to minimize dilution effects).

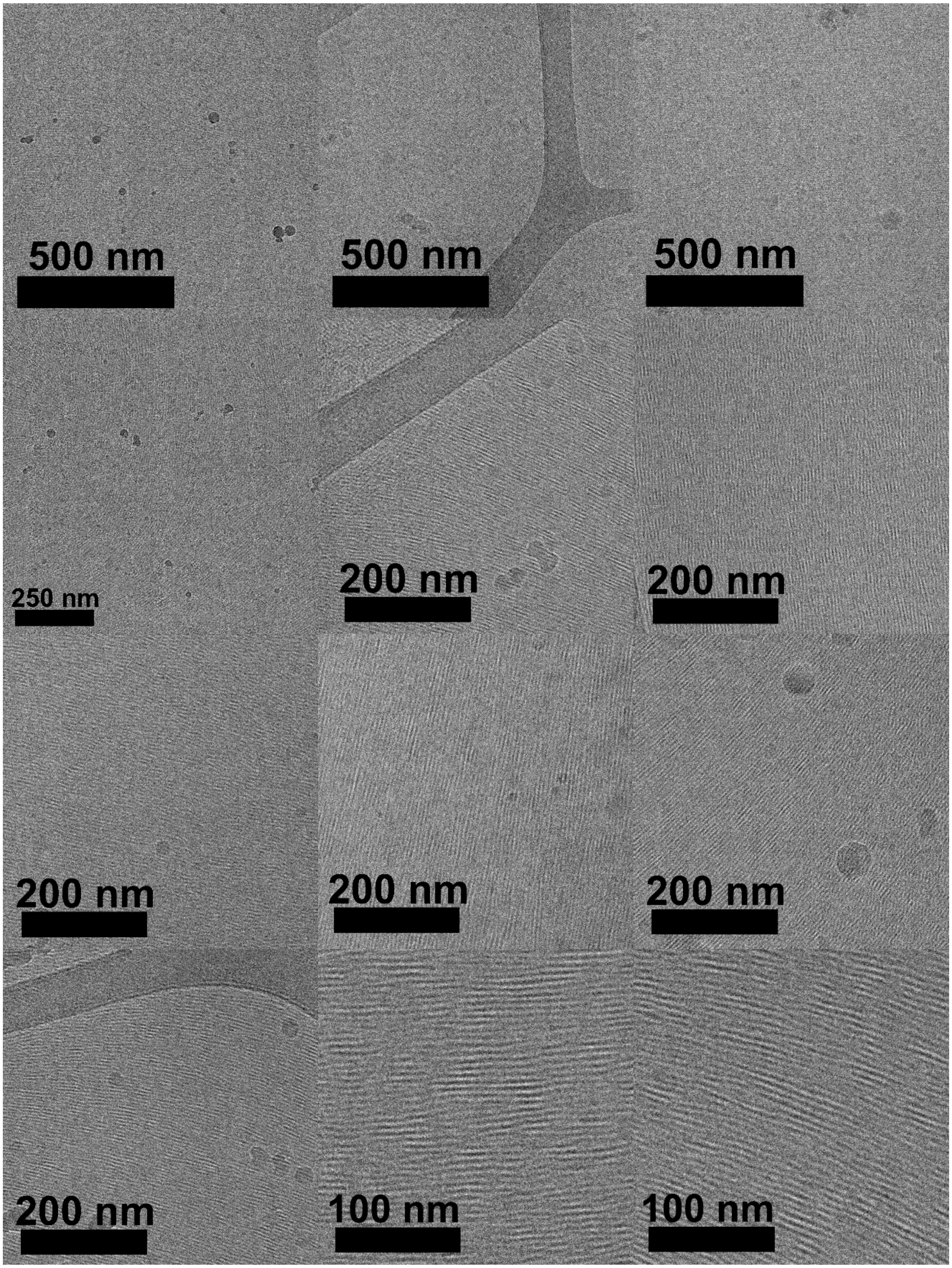

Figure S-11: CryoTEM of SC+6_2A prepped at 20w/w% in MilliQ.

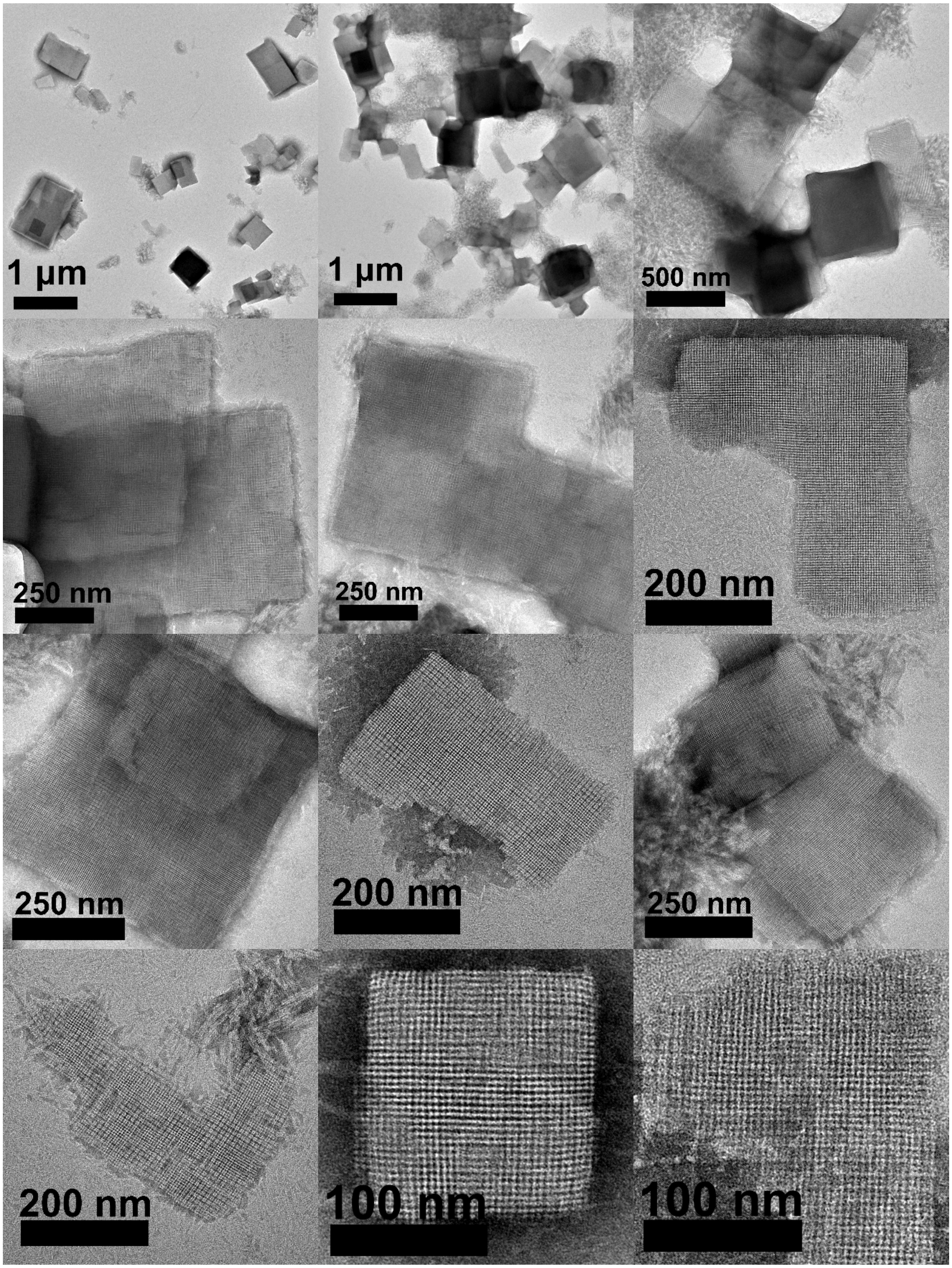

Figure S-12: Negative Stain TEM of SC+6_2A at 5w/w% in 1M NaCl (diluted on grid to minimize dilution effects).

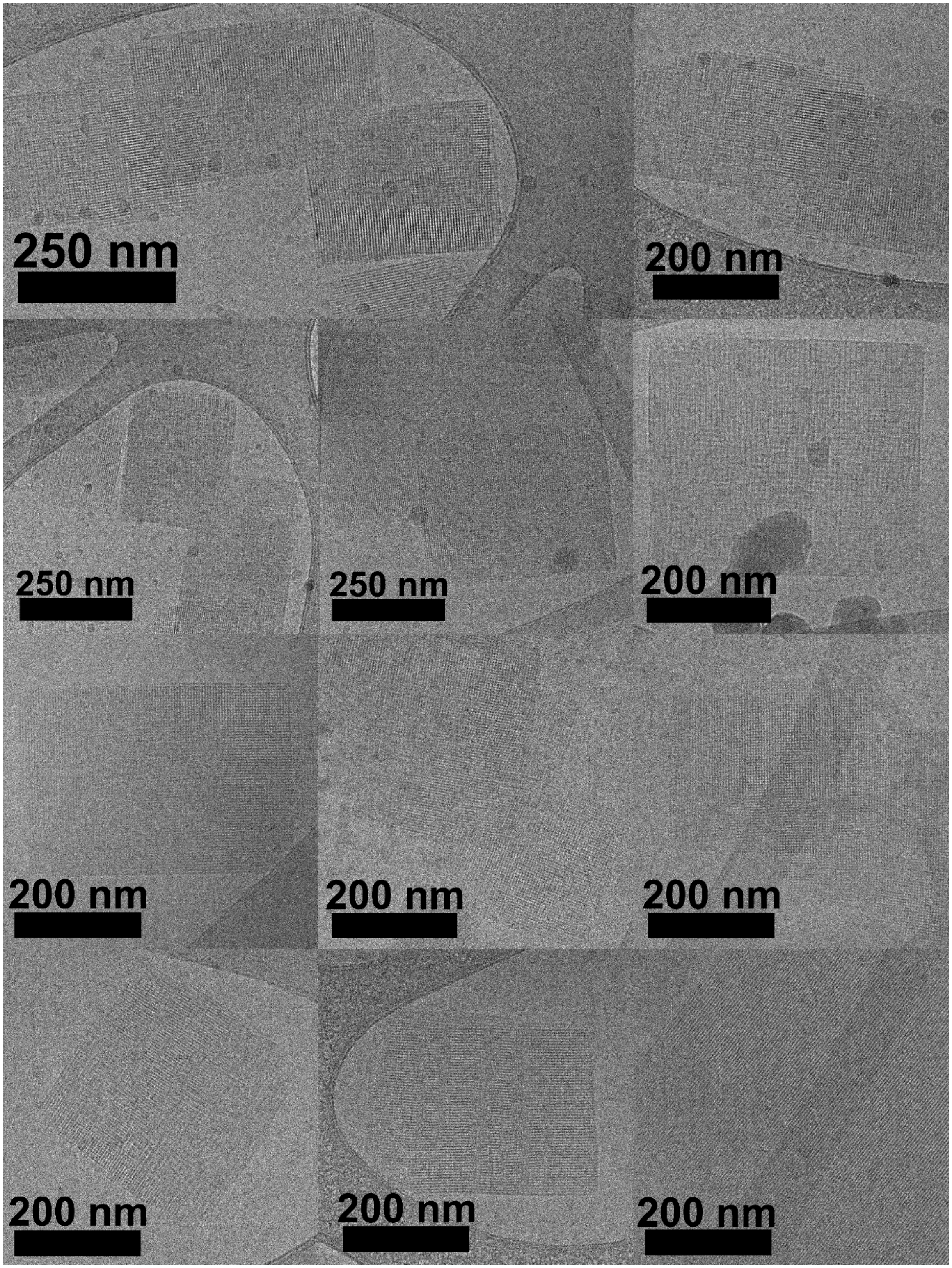

Figure S-13: Cryo-TEM of SC+6_2A at 5w/w% in 1M NaCl.

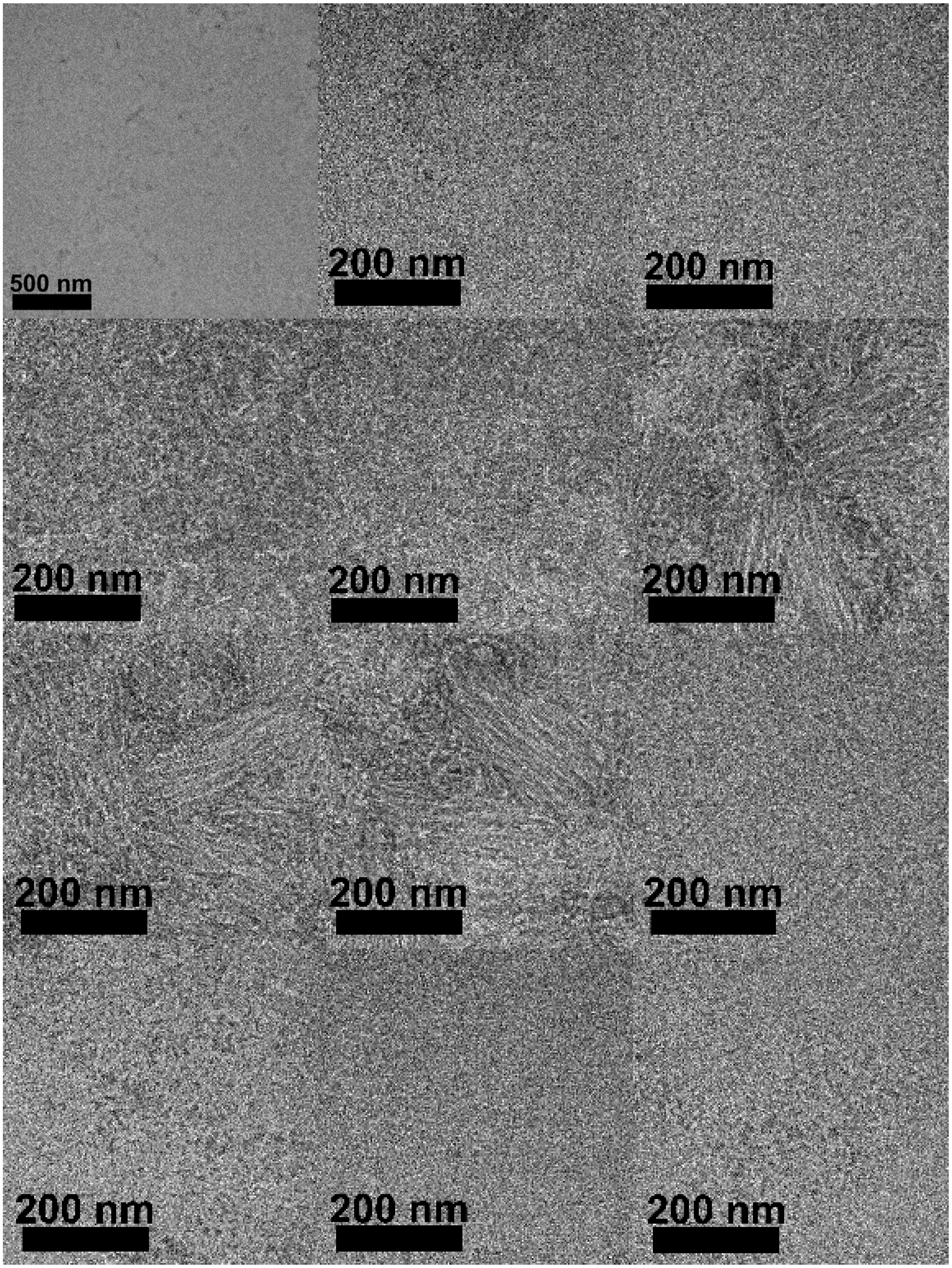

Figure S-14: Negative Stain TEM of SC+6_2A at 10w/w% in 0.2M NaCl aged 1 day.

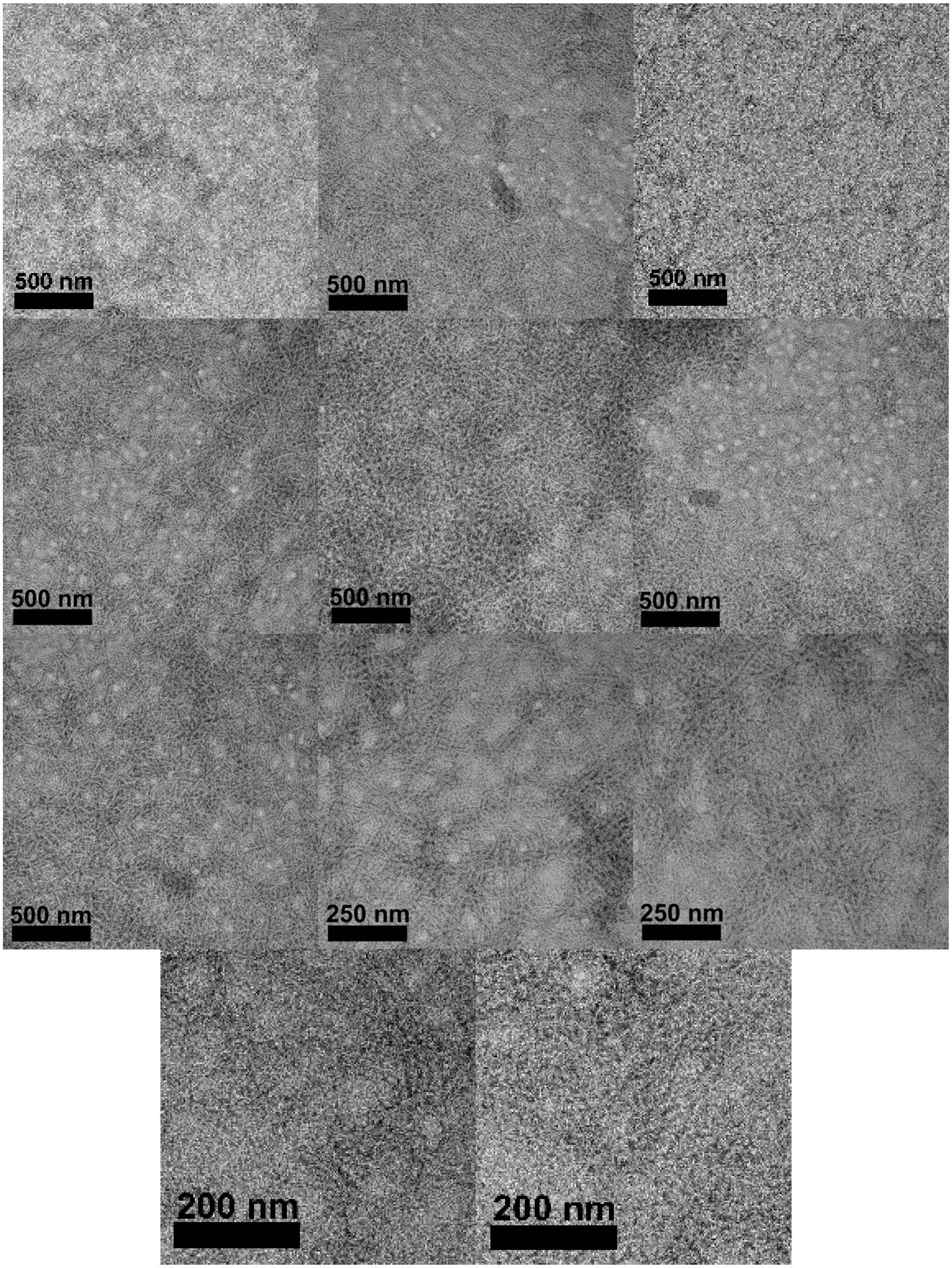

Figure S-15: Negative Stain TEM of SC+6_2A at 10w/w% in 0.2M NaCl aged 1 month

V. Supplemental Small Angle X-ray Scattering (SAXS)

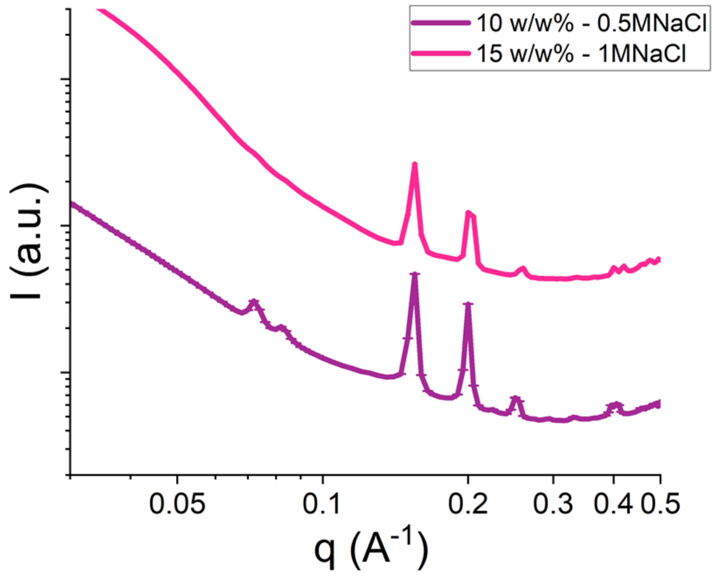

Figure S-16: Small angle x-ray scattering (SAXS) of SC+6_2A at 10 w/w% in 0.5M NaCl and 15 w/w% in 1M NaCl. Both data sets were acquired at Brookhaven National Laboratory.

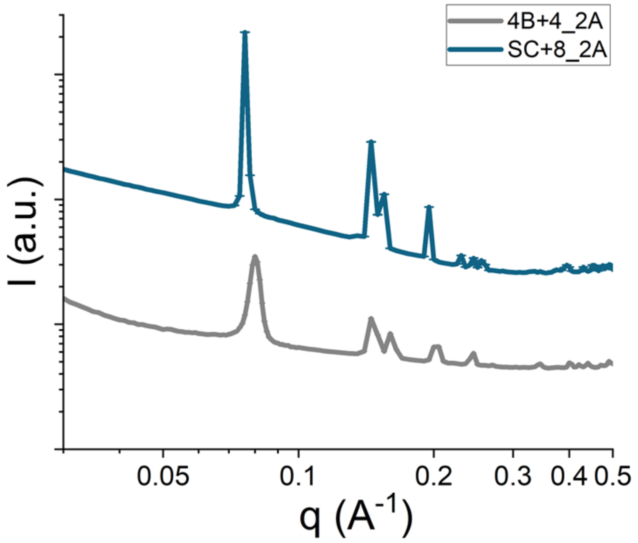

Figure S-17: Small angle x-ray scattering (SAXS) of SC+8_2A at 5 w/w% in 0.5M NaCl and 4B+4_2A at 5 w/v% in 50 mM phosphate buffer. Both data sets were acquired at Brookhaven National Laboratory.

VI. Data Comparisons

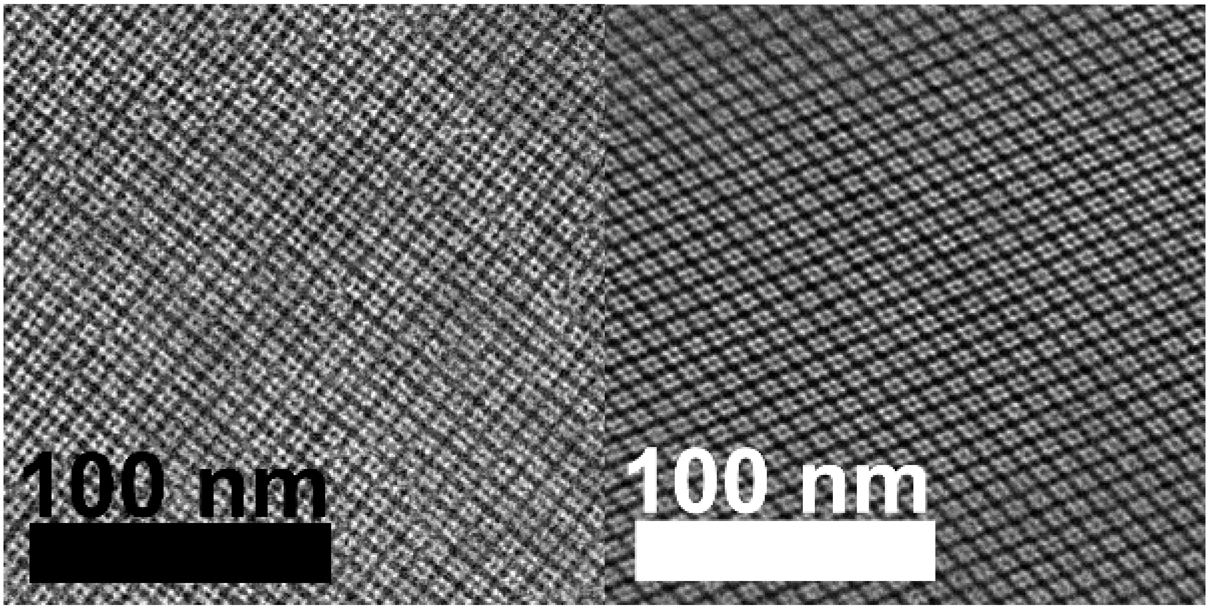

Figure S-18: Negative Stain TEM of (A) SC+8_2A at 5w/w% in 1 M NaCl and (B) 4B+4_2A at 5 w/w% in 50 mM Phosphate Buffer

VII. Lattice Packing Models

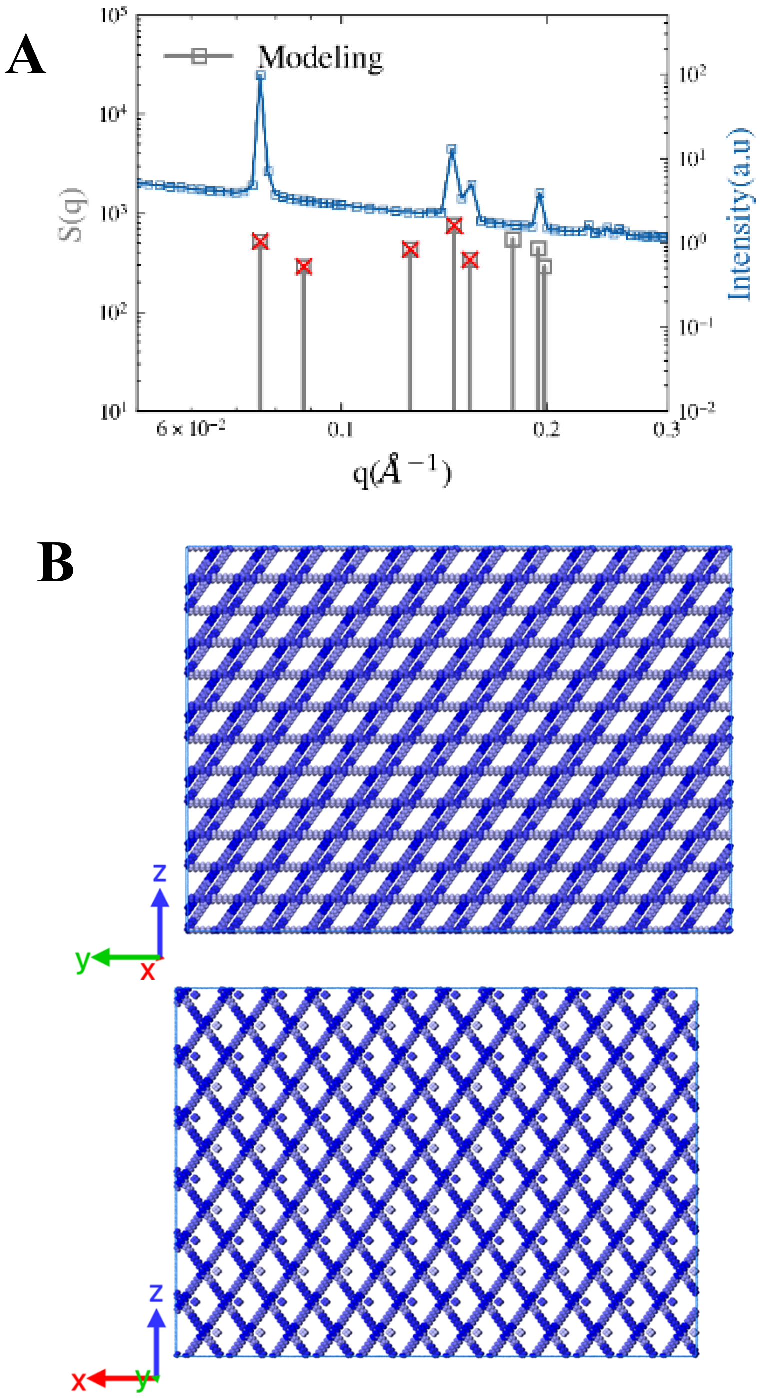

Figure S-19: (A) Comparison of experimental results from SAXS for SC+8_2A to the predicted coarse-grained model of the assembled lattices. The modelled structure factor was calculated for a crystal with cubic dimensions (x, y, and z are equal in length). (B) Additional projections of the proposed lattice structure for SC+8_2A.

A

B

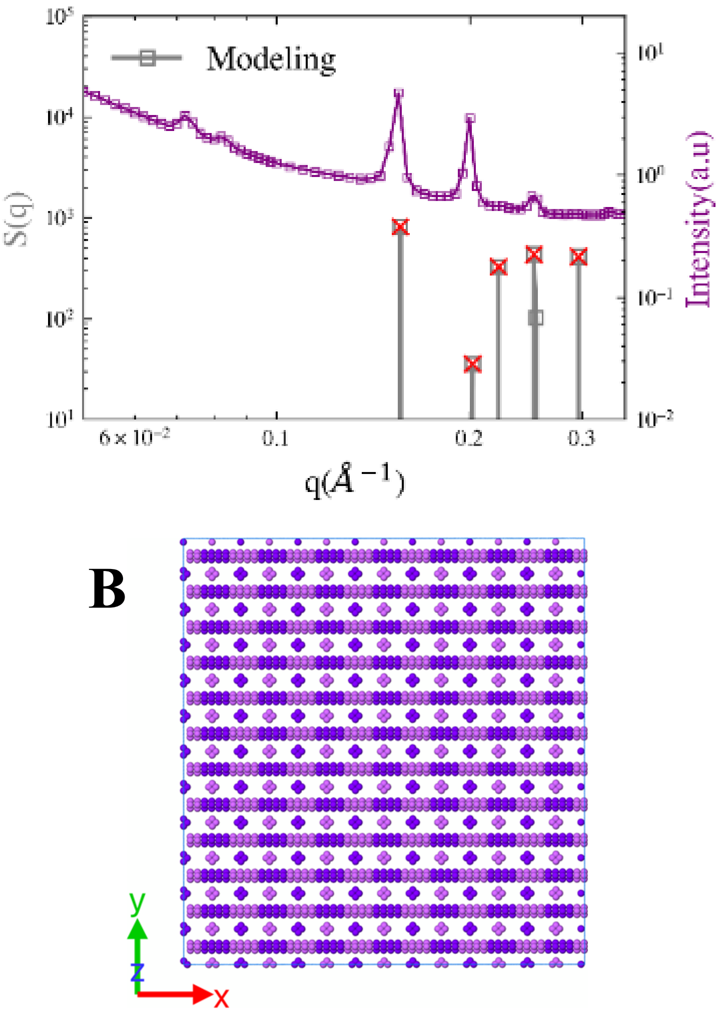

Figure S-20: (A) Comparison of experimental results from SAXS for SC+6_2A to the predicted coarse-grained model of the assembled lattices. The modelled structure factor was calculated for a crystal with cubic dimensions (x, y, and z are equal in length). (B) Additional projection of the proposed lattice structure for SC+6_2A.
